# Hypoxia Drives Material‐Induced Heterotopic Bone Formation by Enhancing Osteoclastogenesis via M2/Lipid‐Loaded Macrophage Axis

**DOI:** 10.1002/advs.202207224

**Published:** 2023-03-27

**Authors:** Dan Li, Yucan Jiang, Ping He, Yeming Li, Yan Wu, Wei Lei, Nanxin Liu, Joost D. de Bruijn, Hua Zhang, Hongmei Zhang, Ping Ji, Huipin Yuan, Mingzheng Li

**Affiliations:** ^1^ Chongqing Key Laboratory of Oral Diseases and Biomedical Sciences Chongqing Municipal Key Laboratory of Oral Biomedical Engineering of Higher Education Stomatological Hospital of Chongqing Medical University Chongqing 401120 P. R. China; ^2^ School of Engineering and Materials Science Queen Mary University of London London E1 4NS UK; ^3^ Kuros Biosciences BV Prof. Bronkhorstlaan 10 Bilthoven 3723 MB The Netherlands; ^4^ Department of Obstetrics and Gynecology The First Affiliated Hospital of Chongqing Medical University Chongqing 400015 P. R. China; ^5^ Huipin Yuan's Lab Chengdu 610000 P. R. China

**Keywords:** heterotopic ossification, hypoxia, macrophage polarization, osteoclast, osteoinductive materials

## Abstract

Heterotopic ossification (HO) is a double‐edged sword. Pathological HO presents as an undesired clinical complication, whereas controlled heterotopic bone formation by synthetic osteoinductive materials shows promising therapeutic potentials for bone regeneration. However, the mechanism of material‐induced heterotopic bone formation remains largely unknown. Early acquired HO being usually accompanied by severe tissue hypoxia prompts the hypothesis that hypoxia caused by the implantation coordinates serial cellular events and ultimately induces heterotopic bone formation in osteoinductive materials. The data presented herein shows a link between hypoxia, macrophage polarization to M2, osteoclastogenesis, and material‐induced bone formation. Hypoxia inducible factor‐1*α* (HIF‐1*α*), a crucial mediator of cellular responses to hypoxia, is highly expressed in an osteoinductive calcium phosphate ceramic (CaP) during the early phase of implantation, while pharmacological inhibition of HIF‐1*α* significantly inhibits M2 macrophage, subsequent osteoclast, and material‐induced bone formation. Similarly, in vitro, hypoxia enhances M2 macrophage and osteoclast formation. Osteoclast‐conditioned medium enhances osteogenic differentiation of mesenchymal stem cells, such enhancement disappears with the presence of HIF‐1*α* inhibitor. Furthermore, metabolomics analysis reveals that hypoxia enhances osteoclastogenesis via the axis of M2/lipid‐loaded macrophages. The current findings shed new light on the mechanism of HO and favor the design of more potent osteoinductive materials for bone regeneration.

## Graphical abstract

Hypoxia enhances macrophage polarization to M2, stimulates lipid loading into M2 macrophage to facilitate osteoclastogenesis, factors generated in osteoclastogenesis initiate heterotopic bone formation. On the contrary, via the axis of M2/lipid‐loaded macrophages, HIF‐1*α* inhibitors inhibit osteoclastogenesis, material‐induced heterotopic bone formation is blocked once osteoclasts are deleted.

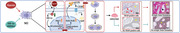

## 1 Introduction

Bone is a hard tissue that makes up the body's skeleton.^[^ ^1^ ^]^ Bone is also a living tissue that is being remodeled continuously, being resorbed by osteoclasts, and being formed by osteoblasts.^[^ ^2^ ^]^ Bone has the ability to repair itself, as long as the extent of the defect dimensions are not over a critical size limit.^[^ ^3^, ^4^ ^]^

Under certain circumstances, bone tissue can be formed in non‐osseus sites in a process that is known as heterotopic ossification (HO), which can either be a hereditary or acquired disease.^[^ ^5^, ^6^ ^]^ Among acquired HO, pathological heterotopic bone formation is often seen after musculoskeletal trauma,^[^ ^7^ ^]^ spinal cord injury,^[^ ^8^ ^]^ central nervous system injury,^[^ ^9^ ^]^ atherosclerotic carotid arteries,^[^ ^10^ ^]^ cardiac valves,^[^ ^11^ ^]^ and in different types of tumors.^[^ ^12^ ^]^ Although these occurrences of pathological HO are an undesired clinical complication, controlled bone formation in non‐osseous sites can be induced by osteoinductive materials without the help of exogenous growth factors/osteogenic cells.^[^ ^13^ ^]^ With fewer disadvantages than other approaches (e.g., autografts,^[^ ^14^, ^15^ ^]^ allografts,^[^ ^15^ ^]^ and engineering bone with growth factors/osteogenic cells^[^ ^16^, ^17^ ^]^), osteoinductive materials hold promises for bone regeneration in situations where bone formation far from the host bone bed is necessary to restore the function of skeletal system (e.g., repair of critical‐sized bone defects and spinal fusion).^[^ ^18^, ^19^, ^20^ ^]^ However, the exact mechanism of material‐induced heterotopic bone formation is not fully known yet.

Acquired HO is often associated with inflammation.^[^ ^21^ ^]^ Governed by gene mutations, hereditary HO is facilitated with inflammation.^[^ ^22^ ^]^ Inflammation is a common feature of HO, and macrophages are critical mediators of inflammation.^[^ ^23^ ^]^ Given the high plasticity, macrophages can acquire different functional phenotypes (e.g., M1 macrophages and M2 macrophages). Increasing evidence indicates that M2 macrophages are indeed relevant for HO.^[^ ^23^ ^]^ It has been suggested that by secreting growth factors, such as the transforming growth factor‐*β*1, bone morphogenetic protein (BMP), activin A, oncostatin M, substance P, neurotrophin‐3, and vascular endothelial growth factor (VEGF), M2 macrophages support angiogenesis and mesenchymal stem cells (MSCs) ‐mediated bone formation by promoting MSCs chondrogenic/osteogenic differentiation in HO.^[^ ^23^, ^24^ ^]^

It appeared that hypoxia played critical roles in heterotopic bone formation.^[^ ^25^ ^]^ On the one hand, hypoxic microenvironment could be created in all cases of acquired HO and HIF‐1*α* was often up‐regulated in heterotopic bone formation, together with the up‐regulation of factors implicating ectopic bone formation, such as BMP, VEGF, and neuropilin‐1.^[^ ^25^, ^26^ ^]^ On the other hand, HIF‐1*α* inhibitors prevented trauma‐induced HO.^[^ ^27^ ^]^ Hypoxia does somehow inhibit osteogenesis of MSCs^[^ ^28^ ^]^ but does enhance angiogenesis.^[^ ^29^, ^30^ ^]^ Angiogenesis and osteogenesis are highly interdependent.^[^ ^31^ ^]^ Angiogenesis is thus suggested as one of the mechanisms that hypoxia plays its role in HO. Hypoxia is a driver of macrophage polarization.^[^ ^32^ ^]^ Macrophages are thought to be the mediator between hypoxia and angiogenesis, with hypoxia modifying macrophage polarization toward M2 macrophages to produce growth factors such as VEGF for angiogenesis.^[^ ^32^, ^33^, ^34^ ^]^ It could be concluded from the available knowledge that angiogenesis and M2 macrophages induced by hypoxia are the possible initiators of pathological HO.

Surgical implantation of osteoinductive materials shares similar responses with HO caused by trauma and injuries. Similar to HO caused by trauma and injuries, inflammation and hypoxia were accompanied with the implantation of materials. Notably, macrophages have also been shown to be involved in material‐induced bone formation. Depletion of macrophages with clodronate liposomes blocked material‐induced bone formation^[^ ^35^ ^]^ and osteoinductive materials favored macrophage polarization to M2.^[^ ^35^, ^36^, ^37^ ^]^ Besides macrophage polarization, the formation of osteoclasts (osteoclastogenesis) has also been shown to be involved in material‐induced bone formation. The role of osteoclastogenesis in material‐induced heterotopic bone formation by materials was not only demonstrated through the presence of osteoclasts prior to material‐induced bone formation,^[^ ^38^, ^39^ ^]^ but was also supported by the observations that osteoinductive materials favored osteoclastogenesis in vitro^[^ ^40^ ^]^ and in vivo.^[^ ^39^, ^41^ ^]^ Meanwhile, osteoinductive materials cannot induce heterotopic bone formation in animals that did not support osteoclast formation in osteoinductive materials.^[^ ^42^ ^]^ Moreover, MSCs seeded on calcium phosphate materials mediated inflammation toward osteoclastogenesis to allow a more robust fashion and to a greater extent bone formation than materials alone, and inhibition of osteoclastogenesis by injection of anti‐RANKL blocked bone formation in MSC‐loaded calcium phosphate material in a similar intramuscular model.^[^ ^43^, ^44^ ^]^ Furthermore, material‐induced heterotopic bone formation was blocked once osteoclasts were deleted.^[^ ^37^, ^40^ ^]^ These findings demonstrated an important role of osteoclastogenesis in material‐induced bone formation. More interestingly, it appeared that the role of M2 macrophage in material‐induced heterotopic bone formation is mediated by osteoclastogenesis, since osteoclastogenesis occurred in between macrophage polarization to M2 and formation of bone in osteoinductive materials^[^ ^39^ ^]^ and macrophages could fuse to form osteoclasts.^[^ ^45^ ^]^

Considering the above‐mentioned findings, we speculate that the degree of hypoxia varies with osteoinductive material implants and non‐osteoinductive material implants, and such a difference subsequently affects macrophage polarization, angiogenesis, osteoclastogenesis, and finally material‐induced bone formation. To demonstrate the hypothesis, material‐induced bone formation was verified first with two CaP ceramics having different physicochemical properties. An osteoinductive calcium phosphate ceramic with submicron needle‐like surface morphology (MG) and non‐osteoinductive tricalcium phosphate ceramics with micron surface morphology (TCPB)^[^ ^46^ ^]^ in FVB mouse intramuscular implantation model, the difference of hypoxic status in MG and TCPB was also illustrated. The role of hypoxia in material‐induced bone formation was evaluated with HIF‐1*α* inhibitor (rapamycin and PX‐478) and HIF‐1*α* activator (DFO) in vivo. How hypoxia plays its role in material‐induced bone formation (e.g., angiogenesis, macrophage polarization, and osteoclastogenesis) was then subjected to exploration in both in vivo studies with different time points and in vitro studies with the culture of bone marrow‐derived macrophages (mBMDMs) with or without the interference of HIF‐1*α* inhibitors (rapamycin). Finally, the suggestive mechanism by which hypoxia plays its role in material‐induced bone formation was initially investigated with MSCs (CRL‐12424) culture in vitro.

## 2 Results

### 2.1 MG But Not TCPB Induces Bone Formation Following Intramuscular Implantation in FVB Mice

**Figure 1:**
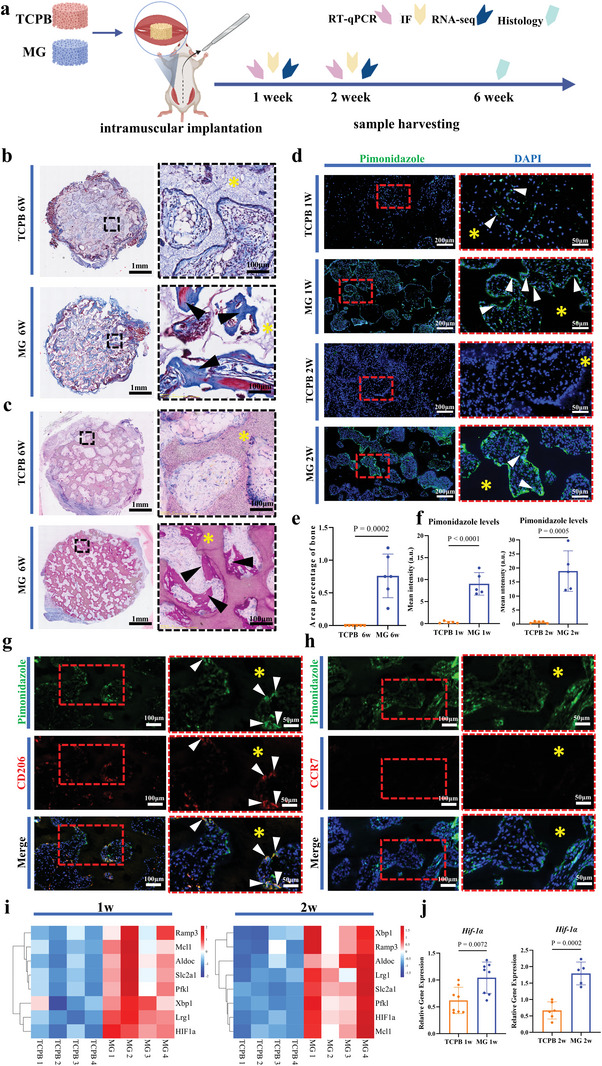
Hypoxia, HIF‐1*α* expression, and CaP‐induced bone formation in TCPB and MG implants. a) Scheme of the study design. b) Histology of samples harvested at week 6 (decalcified sections, Masson's trichrome stain) (*n* = 4). c) Histology of samples harvested at week 6 (non‐decalcified sections, methylene blue/basic fuchsin stain) (*n* = 6). d) Immunofluorescence staining of implants with Pimonidazole (green) and DAPI (blue) staining harvested at week 1 and week 2 (*n* = 5). e) Area percentage of bone in available space of the implants at week 6 (*n* = 6). Two‐tailed Student's *t*‐test. f) Quantification of pimonidazole staining intensity (artificial units, a.u.) in samples harvested at week 1 and week 2 (*n* = 5). Two‐tailed Student's *t*‐test. g) Dual immunofluorescence staining of implants with Pimonidazole (green) and CD206 (red) staining harvested at week 1 (*n* = 4). h) Dual immunofluorescence staining of implants with Pimonidazole (green) and CCR7 (red) staining harvested at week 1 (*n* = 4). i) Expression of hypoxia‐related genes in TCPB and MG implants at week 1 and week 2 (*n* = 4). j) The column diagrams showing the gene expression of *Hif‐1α* in TCPB and MG implants at week 1 and week 2 (*n* = 5–8). Two‐tailed Student's *t*‐test. The yellow asterisks indicate the materials, the black arrows indicate new bone, the white arrows indicate pimonidazole‐positive cells in (d), the white arrows indicate pimonidazole‐positive cells or CD206‐positive cells in (g). Each error bar represents the mean ± SD. Differences were considered statistically significant at *p* < 0.05.

To evaluate material‐induced bone formation in FVB/NCrl (FVB) mice, both TCPB and MG discs (5.0 × 2.5 mm) were implanted in gluteal muscle of FVB mice for 6 weeks (**Figure** **1**a). To confirm bone formation, samples were subjected to both decalcified sections stained with Masson's trichrome (*n* = 4 per material) and non‐decalcified section stained with methylene blue/basic fuchsin (*n* = 6 per material). Bone was detected inside all MG implants in both decalcified sections (Figure 1b) and non‐decalcified sections (Figure 1c) but no bone formation was observed in any TCPB implants. Quantitatively, bone was found in 21 out of the total 39 non‐decalcified MG sections and bone was not observed in any of the non‐decalcified TCPB sections (Figure 1e; Figure S4e, Supporting Information); the area percentage of bone in available space in MG implants was 0.76 ± 0.33% (Figure 1e; Figure S4e, Supporting Information).

### 2.2 MG Implants Are More Hypoxic and Exhibit Up‐Regulation of HIF‐1*α*

To investigate hypoxia and expression of HIF‐1*α* in MG and TCPB following intramuscular implantation in mice, samples were collected at week 1 and week 2 after implantation. Pimonidazole, a chemical which is reductively activated in hypoxic cells and forms stable adducts with thiol (sulfhydryl) groups in proteins, peptides, and amino acids,^[^ ^47^ ^]^ was injected intraperitoneally 1 h prior to sample harvesting. Immunofluorescence staining of pimonidazole showed a higher pimonidazole level in MG than TCPB at week 1 and week 2, with an increase from week 1 to week 2 in MG implants (*n* = 5 per time point per material, Figure 1d,f). Double immunofluorescence staining showed the co‐existence of ARG‐1‐positive cells and pimonidazole‐positive cells in MG implants at week 1 (*n* = 4 per time point per material, Figure 1g) but no co‐existence of CCR7‐positive cells and pimonidazole‐positive cells (*n* = 4 per time point per material, Figure 1h). Transcriptomic analysis showed significant up‐regulation of hypoxia‐related genes (e.g., *Aldoc*, *Xbp1*, *Ramp3*, *Slc2a1*, *Pfk1*, *Mcl1*, *Lrg1*, *Hif‐1α*) in MG implants compared to TCPB implants (*n* = 4 per time point per material, Figure 1i). As shown in real‐time quantitative polymerase chain reaction (RT‐qPCR), gene expression of *Hif‐1α* was significantly up‐regulated in MG at week 1 and week 2 (*n* = 5–8 per time point per material, Figure 1j).

### 2.3 Vessel Formation Shows No Difference in MG and TCPB Implants

To investigate the role of angiogenesis in material‐induced bone formation, MG and TCPB were intramuscularly implanted in mice for 1 week and 2 weeks (Figure S2a, Supporting Information). Difference of angiogenesis‐related gene expression (e.g., *Vegfb*, *Angpt4*, *Egf*, *Vegfc*, *Vegfd*, *Angpt1*, *Pecam1*, *Emcn*, *Eng*) was not detected at either week 1 or week 2 in transcriptomic analysis (*n* = 4 per time point per material, Figure S2c, Supporting Information). To further verify the results of transcriptomic analysis, RT‐qPCR was performed, and no difference in angiogenesis‐related gene expression (*Cd31*, *Emcn*, and *Vegfa*) between MG and TCPB implants were noted at week 1 and week 2 either (*n* = 6–7, per time point per material, Figure S2d, Supporting Information). In addition, immunofluorescence staining showed that number of vessels [marked with CD31, *α*‐Smooth Muscle Actin (*α*‐SMA) and Endomucin(EMCN)] in MG was visually similar to that in TCPB at week 1 and week 2 (*n* = 4 per time point per material, Figure S2b, Supporting Information).

### 2.4 HIF‐1*α* Inhibitors Inhibit Macrophage Polarization toward M2 and Osteoclastogenesis in MG Implants

**Figure 2:**
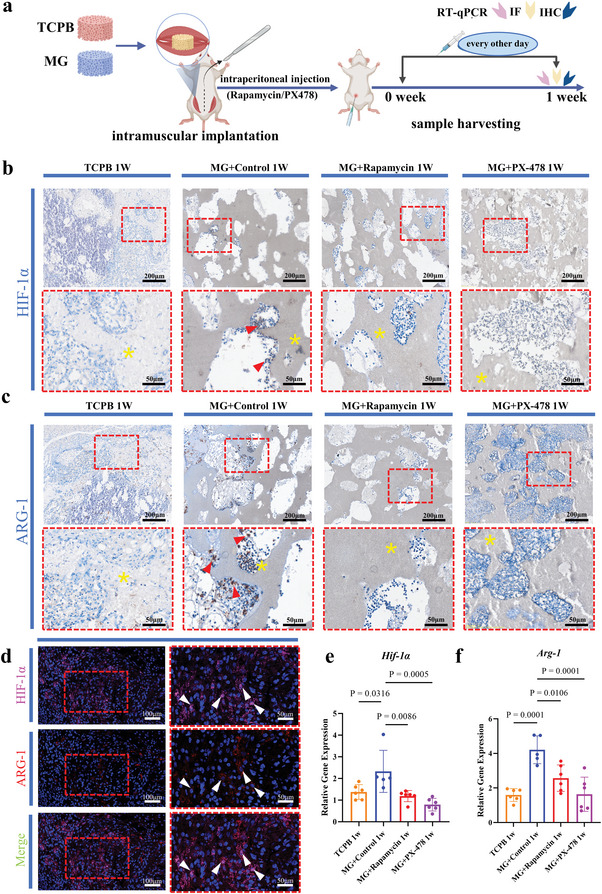
Influence of HIF‐1*α* inhibitors (rapamycin and PX‐478) on expression of HIF‐1*α* and macrophage polarization in MG and TCPB implants at week 1. a) Scheme of the study design. b) HIF‐1*α* immunohistochemical staining (*n* = 4). c) ARG‐1 immunohistochemical staining (*n* = 4). d) ARG‐1 and HIF‐1*α* dual immunofluorescence staining in MG at week 1 (*n* = 4). e) Gene expression of *Hif‐1α* (*n* = 5–6). One‐way ANOVA with Tukey's post‐test. f) Gene expression of *Arg‐1* (*n* = 5–6). One‐way ANOVA with Tukey's post‐test. The yellow asterisks indicate the materials, the red triangles indicate ARG‐1 or HIF‐1*α* positive cells with immunohistochemical staining, the white triangles indicate ARG‐1 or HIF‐1*α* positive cells with immunofluorescence staining. Each error bar represents the mean ± SD. Differences were considered statistically significant at *p* < 0.05.

**Figure 3:**
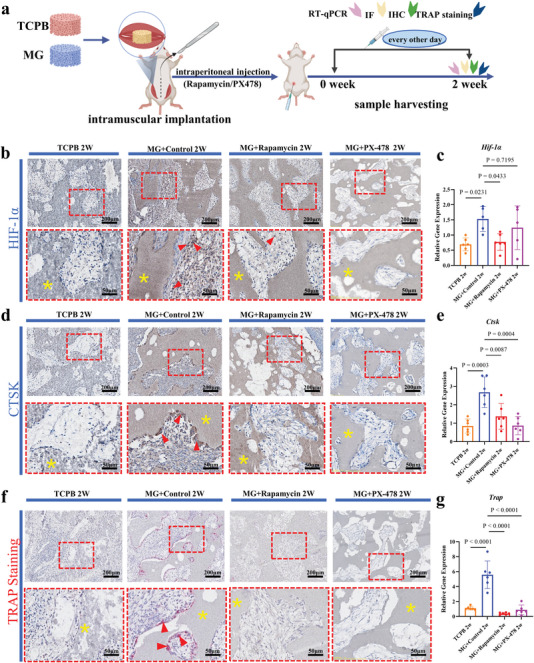
Influence of HIF‐1*α* inhibitors (rapamycin and PX‐478) on HIF‐1*α* and osteoclastogenesis in MG implants at week 2. a) Scheme of the study design. b) HIF‐1*α* immunohistochemical staining (*n* = 4). c) *Hif‐1α* genes expression (*n* = 5–6). One‐way ANOVA with Tukey's post‐test. d) CTSK immunohistochemical staining (*n* = 4). e) *Ctsk* gene expression (*n* = 6). One‐way ANOVA with Tukey's post‐test. f) TRAP staining of TCPB and MG implants (*n* = 4). g) *Trap* gene expression (*n* = 6). One‐way ANOVA with Tukey's post‐test. The yellow asterisks indicate the materials, the red triangles indicate HIF‐1*α*, CTSK, or TRAP positive cells. Each error bar represents the mean ± SD. Differences were considered statistically significant at *p* < 0.05.

To investigate the effect of HIF‐1*α* on macrophage polarization and osteoclastogenesis in vivo, rapamycin and PX‐478, which have been shown to inhibit HIF‐1*α* transcription,^[^ ^48^, ^49^ ^]^ were used. Mice bearing MG implants received intraperitoneal injections every other day for 1 week or 2 weeks, the implants were evaluated with tartrate‐resistant acid phosphatase (TRAP) staining, immunohistochemical staining and gene analysis (**Figures** **2**a and **3**a), with TCPB as the negative control.

Fewer HIF‐1*α*‐positive cells, fewer Arginase 1 (ARG‐1)‐positive cells and fewer F4/80‐positive cells were observed in TCPB implants and MG implants treated with rapamycin and PX‐478 at week 1, compared with MG controls (*n* = 4 per group, Figure 2b,c; Figure S3b, Supporting Information). Fewer CCR7‐positive cells were observed in TCPB implants and MG implants at week 1, regardless the presence of rapamycin and PX‐478 (*n* = 4 per group, Figure S3a, Supporting Information). Meanwhile, gene expression of *Hif‐1α* and *Arg‐1* was significantly down‐regulated in TCPB group and MG groups treated with rapamycin and PX‐478 at week 1 (*n* = 5–6 per group, Figure 2e,f). Furthermore, double immunofluorescence staining showed the co‐existence of ARG‐1‐positive cells and HIF‐1*α*‐positive cells in MG implants at week 1 (*n* = 4, Figure 2d).

Similarly, fewer HIF‐1*α*‐positive cells and fewer Cathepsin K (CTSK)‐positive cells were seen at week 2 in TCPB implants and MG implants treated with rapamycin and PX‐478, compared to MG controls (*n* = 4 per group, Figure 3b,d). TRAP‐positive cells were hardly seen in MG rapamycin group, MG PX‐478 group and TCPB group at week 2 (*n* = 4 per group, Figure 3f). In agreement with the histological analysis, significantly less *Hif‐1α*, *Trap*, and *Ctsk* gene expression was detected in MG rapamycin group, MG PX‐478 group and TCPB group at week 2, compared to MG controls (*n* = 5–6 per group, Figure 3c,e,g).

### 2.5 Pharmacologic Regulating HIF‐1*α* Affects Material‐Induced Bone Formation

**Figure 4:**
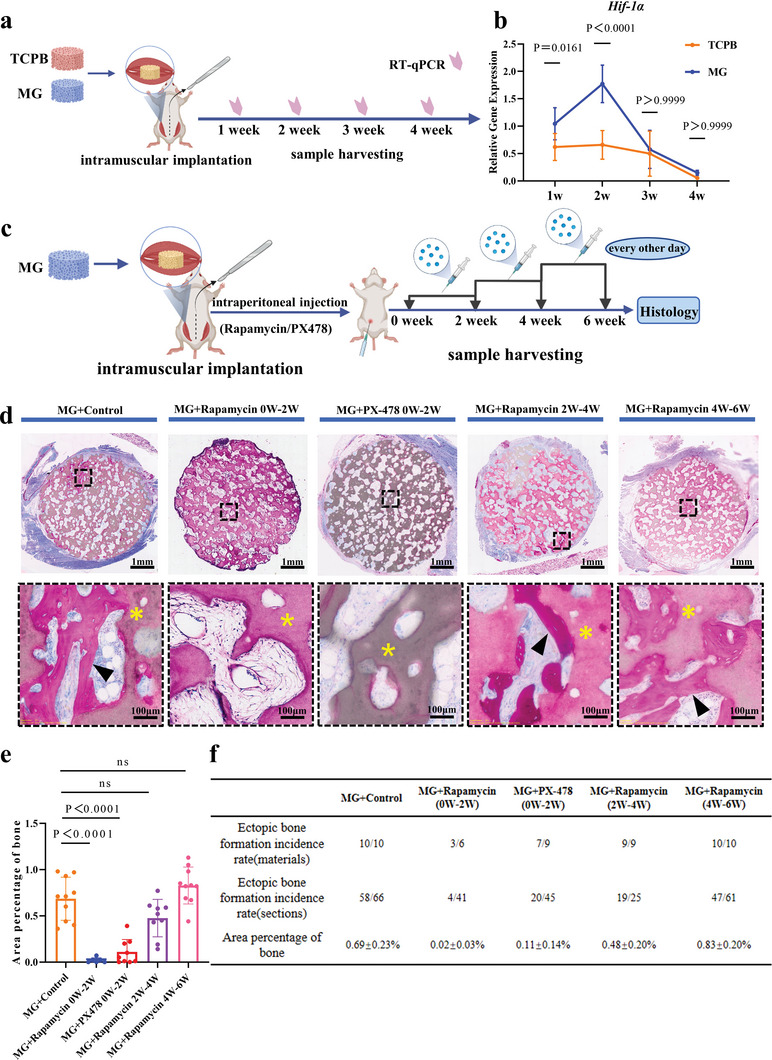
Time‐dependent HIF‐1*α* activation and influence of HIF‐1*α* inhibitors on bone formation following intraperitoneal injection. a,c) Scheme of the study design. b) Time‐dependent *Hif‐1α* gene expression in TCPB and MG implants (*n* = 5–8). Two‐way ANOVA with Bonferroni's multiple comparisons test. d) Histology of samples harvested at week 6 (non‐decalcified sections, methylene blue/basic fuchsin stain) (*n* = 6–10). e) Area percentage of bone in available space of the implants harvested at week 6 (*n* = 6–10). One‐way ANOVA with Tukey's post‐test. f) A summary of bone formation at week 6 (*n* = 6–10). The yellow asterisks indicate the materials, the black arrows indicate new bone. Each error bar represents the mean ± SD. Differences were considered statistically significant at *p* < 0.05.

To investigate the influence of HIF‐1*α* on material‐induced bone formation, both HIF‐1*α* inhibitors (rapamycin and PX‐478) and HIF‐1*α* activator (desferrioxamine, DFO^[^ ^50^ ^]^), were applied to mice bearing implants via either intraperitoneal (**Figure** **4**c) or peri‐implant injection (Figure S4a, Supporting Information) at early time or later time. For early time treatment, chemicals were applied every other day in the first 2 weeks. For later time treatment, chemicals were injected every other day from week 2 to week 4 or from week 4 to week 6. PBS was injected as the control, if necessary, samples were harvest at week 6 for histological evaluation and subjected to histological and histomorphometrical assays using non‐decalcified sections. At the meantime, HIF‐1*α* activation in TCPB and MG implants were evaluated by week from week 1 to week 4 with respect to gene expression (Figure 4a). *Hif‐1α* gene expression was up‐regulated in MG implants compared to TCPB implants at both week 1 and week 2, then down‐regulated thereafter, resulting no difference between MG implants and TCPB implants at week 3 and week 4 (*n* = 5–8 per time point per material, Figure 4b). Bone formation in implants was up to not only the chemical treatments but also the time the treatments applied. When intraperitoneally injected between 0–2 week, Rapamycin and PX‐478 significantly inhibited bone formation in MG implants at week 6 (*n* = 6–10 per group, Figure 4d–f); when intraperitoneally injected between 2–4 week and 4–6 week, Rapamycin did not inhibit bone formation (*n* = 9–10 per group, Figure 4d–f). Peri‐implant injection of rapamycin and PX‐478 between 0–2 week inhibited bone formation in MG implants at week 6 (*n* = 10–13 per group, Figure S4b–d, Supporting Information), while peri‐implant injection of DFO between 0–2 week enhanced bone formation in MG implants (*n* = 6 per group, Figure S4b–d, Supporting Information). Peri‐implant injection of DFO made it possible to form bone in TCPB implants at week 6 (Figure S4b, Supporting Information), with bone incidence of 7/8 (7 out of 8 TCPB implants at week 6) and the area percentage of bone in available space in TCPB implants was 0.07 ± 0.11% (Figure S4d, Supporting Information). Intraperitoneal injection of PBS did not affect bone formation in MG implants at week 6 (0.76 ± 0.33% vs 0.69 ± 0.23%; Figure 1e, Figure S4e, Supporting Information, and Figure 4e,f), while peri‐implant injection of PBS increased bone formation in MG implants at week 6 (0.76 ± 0.33% vs 1.32 ± 0.29%; Figure 1e, Figure S4e, Supporting Information, and Figure S4c,d, Supporting Information).

### 2.6 Pharmacologic Inhibition of HIF‐1*α* Represses Macrophage Polarization toward M2 and Osteoclastogenesis In Vitro

**Figure 5:**
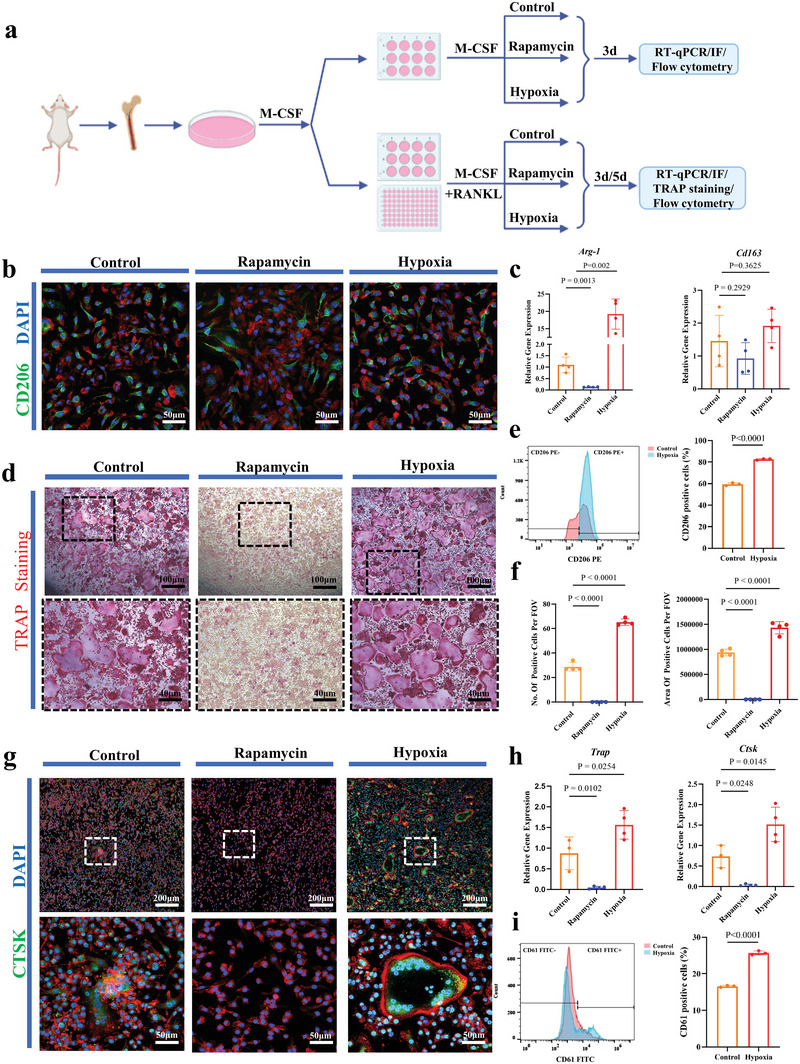
Influence of hypoxia and rapamycin on macrophage polarization and osteoclastogenesis of mBMDMs in vitro. a) Scheme of the study design. b) CD206 immunofluorescence staining after mBMDMs cultured with M‐CSF for 3 days (*n* = 4). c) Gene expression of *Arg‐1* and *Cd163* after mBMDMs cultured with M‐CSF for 3 days (*n* = 4). One‐way ANOVA with Tukey's post‐test. d) TRAP staining after mBMDMs cultured with both M‐CSF and RANKL for 5 days (*n* = 4). e) Flow cytometry showing percentage of CD206‐positive cells after mBMDMs cultured with M‐CSF for 3 days (*n* = 3). Two‐tailed Student's *t*‐test. f) Number and the area of TRAP‐positive cells in mBMDMs cultured with M‐CSF and RANKL for 5 days (*n* = 4). One‐way ANOVA with Tukey's post‐test. g) CTSK immunofluorescence staining after mBMDMs cultured with both M‐CSF and RANKL for 3 days (*n* = 4). h) Gene expression of *Trap* and *Ctsk* after mBMDMs cultured with both M‐CSF and RANKL for 5 days (*n* = 3–4). One‐way ANOVA with Tukey's post‐test. i) Flow cytometry showing percentage of CD61‐positive cells in mBMDMs cultured with M‐CSF and RANKL for 5 days (*n* = 3). Two‐tailed Student's *t*‐test. Each error bar represents the mean ± SD. Differences were considered statistically significant at *p* < 0.05.

The mBMDMs were cultured in vitro with macrophage‐colony stimulating factor (M‐CSF) alone or together with receptor activator of nuclear factor‐*κ*B ligand (RANKL) to explore the factors affecting macrophage polarization and osteoclastogenesis (*n* = 3–4, per case) (**Figure** **5**a). Introducing rapamycin in macrophage polarization culture system significantly down regulated *Arg‐1* gene expression (Figure 5c) and negatively affected the numbers of CD206‐positive cells (Figure 5b) but did not significantly affect *Cd163* gene expression (Figure 5c). Low oxygen significantly up‐regulated *Arg‐1* gene expression in macrophage polarization culture (Figure 5c) but did not have much impact on CD206‐positive cells (Figure 5b) and *Cd163* gene expression (Figure 5c). Furthermore, flow cytometry showed a higher ratio of CD206‐positve cells (Figure 5e) but a lower ratio of CCR7‐positve cells (Figure S6a, Supporting Information) in low oxygen culture with M‐CSF alone for 3 days than in normal oxygen culture. The percentage of CD206‐positive cells was 59.43 ± 1.30% in normal oxygen culture and 82.63 ± 0.64% in low oxygen culture (Figure 5e). The percentage of CCR7‐positive cells was 35.60 ± 1.28% in normal oxygen culture and 29.27± 2.48% in low oxygen culture (Figure S6b, Supporting Information). In vitro osteoclastogenesis evaluation system, rapamycin sharply down regulated both *Trap* gene and *Ctsk* gene expression at day 5 (Figure 5h) and decreased both the number and area of TRAP‐positive cells at day 5 (Figure 5d,f). Low oxygen significantly up‐regulated both *Trap* gene and *Ctsk* gene at day 5 (Figure 5h) and increased both the number and area of TRAP‐positive cells at day 5 (Figure 5d,f). CTSK‐positive cells with large cell bodies were seen under low oxygen at day 3, CTSK signal was observed without typical osteoclast morphology under normal oxygen and no CTSK signal was visible in the case of rapamycin at day 3 (Figure 5g). Flow cytometry showed a higher ratio of CD61 (a marker of osteoclasts)‐positive cells in low oxygen culture with M‐CSF and RANKL for 5 days than in normal oxygen culture (Figure 5i). The percentage of CD61‐positive cells was 16.57 ± 0.25% in normal oxygen culture and 25.70 ± 0.56% in low oxygen culture (Figure 5i). Furthermore, flow cytometry showed rapamycin sharply decreased CD61‐positive cells(Figure S7a, Supporting Information), with 6.94 ± 0.27% of CD61‐positive cells in the case of rapamycin and 16.63 ± 0.21% of CD61‐positive cells in the control (Figure S7b, Supporting Information).

### 2.7 Pharmacologic Inhibition of HIF‐1*α* Affects Metabolomics During Osteoclastogenesis In Vitro

**Figure 6:**
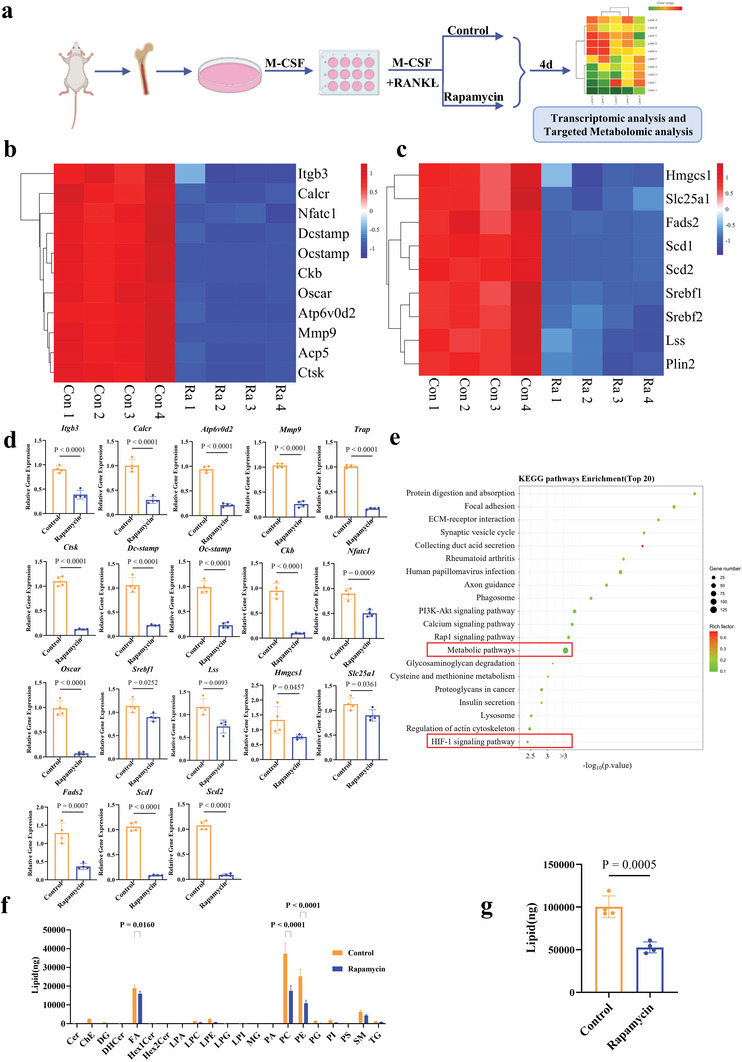
Transcriptomic and targeting metabolomics analysis of osteoclastogenesis culture with/without rapamycin. a) Scheme of the study design. b) Heatmap of osteoclast‐associated genes (*n* = 4). c) Heatmap of lipid accumulation‐related gene (*n* = 4). d) Expression of osteoclast‐associated genes and lipid accumulation‐related genes (*n* = 4). Two‐tailed Student's *t*‐test. e) Enriched KEGG pathway of commonly differentially expressed genes between rapamycin group and control group at day 4 (*n* = 4). f) Lipid class analysis between rapamycin group and control group at day 4 (*n* = 4). Two‐way ANOVA with Bonferroni's multiple comparisons test. g) Total Lipids between rapamycin group and control group at day 4 (*n* = 4). Two‐tailed Student's *t*‐test. Each error bar represents the mean ± SD. Differences were considered statistically significant at *p* < 0.05.

The mBMDMs were cultured with osteoclastogenic medium with/without rapamycin for 4 days to identify the key metabolomics during osteoclastogenesis (*n* = 4, **Figure** **6**a). Transcriptome analysis showed that osteoclastogenesis‐related genes (e.g., *Trap* (*Acp5*), *Ctsk*, *Mmp9*, *Itgb3*, *Nfact1*, *Dc‐stamp*, *Ckb*, *Calcr*, *Atp6v0d2*, *Oc‐stamp, Oscar*) were significantly down‐regulated in the rapamycin group (Figure 6b). Such a trend of gene expression was confirmed by RT‐qPCR (Figure 6d). Meantime, lipid accumulation‐related genes (e.g., *Srebf1*, *Lss*, *Hmgcs1*, *Slc25a1*, *Fads2*, *Scd1*, *Scd2*) were significantly down‐regulated in the rapamycin group (Figure 6c), such a trend of gene expression was confirmed by RT‐qPCR as well (Figure 6d). When genomes (KEGG) analysis was performed with the differentially expressed genes, the related signaling pathways underneath were shown, with metabolic pathway and HIF‐1 signaling pathway significantly down‐regulated in rapamycin group (Figure 6e). A down regulation of total lipids in rapamycin group was shown in targeting metabolomics analysis (Figure 6g). Among all lipids, phosphatidylethanolamine (PE) and phosphatidylcholine (PC) were significantly affected most by rapamycin (Figure 6f).

**Figure 7:**
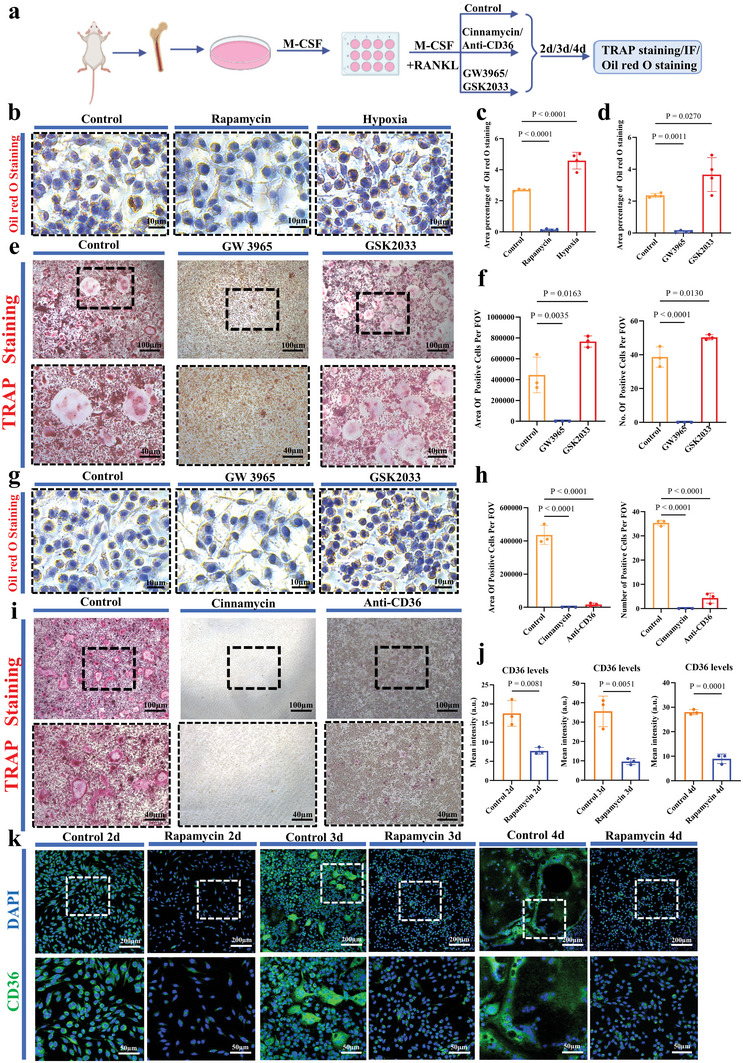
Influence of lipid accumulation on osteoclastogenesis. a) Schematic diagram of the experimental design. b) Oil red O staining of osteoclastogenic culture of mBMDMs treated with rapamycin or hypoxia for 2 days (*n* = 4). c) Area of percentage Oil red O staining of osteoclastogenic culture of mBMDMs treated with rapamycin or hypoxia for 2 days (*n* = 4). One‐way ANOVA with Tukey's post‐test. d) Area of percentage Oil red O staining of osteoclastogenic culture of mBMDMs treated with GW3965 or GSK2033 for 2 days (*n* = 4). One‐way ANOVA with Tukey's post‐test. e) TRAP staining of osteoclastogenic culture of mBMDMs treated with GW3965 or GSK2033 for 4 days (*n* = 3). f) Number and area of TRAP‐positive cells in osteoclastogenic culture of mBMDMs treated with GW3965 or GSK2033 for 4 days (*n* = 3). One‐way ANOVA with Tukey's post‐test. g) Oil red O staining of osteoclastogenic culture of mBMDMs treated with GW3965 or GSK2033 for 2 days (*n* = 4). h) Number and area of TRAP‐positive cells in osteoclastogenic culture of mBMDMs treated with cinnamycin or anti‐CD36 antibody for 4 days (*n* = 3). One‐way ANOVA with Tukey's post‐test. i) TRAP staining of osteoclastogenic culture of mBMDMs treated with cinnamycin or anti‐CD36 antibody for 4 days (*n* = 3). j) Quantification of CD36 staining intensity (artificial units, a.u.) in osteoclastogenesis culture with/without rapamycin at day 2, 3, and 4 (*n* = 3). Two‐tailed Student's *t*‐test. k) CD36 immunofluorescence staining of osteoclastogenesis culture with/without rapamycin at day 2, 3, and 4 (*n* = 3). Each error bar represents the mean ± SD. Differences were considered statistically significant at *p* < 0.05.

When mBMDMs treatment with rapamycin, less lipid droplets (with oil red O staining) were observed in rapamycin group compared to the cultures with the presence of M‐CSF and RANKL at day 2 (*n* = 4 per group, **Figure** **7**b,c), significantly less CD36‐positive cells were observed in rapamycin group at day 2, day 3, and day 4 (*n* = 3 per group, Figure 7k,j). In contrast, low oxygen significantly increased the number and area of lipid droplets at day 2 (*n* = 4 per group, Figure 7b,c). When GW3965, a lipid accumulation inhibitor,^[^ ^51^ ^]^ was introduced into osteoclastogenic culture of mBMDMs (containing both M‐CSF and RANKL) (Figure 7a), it significantly decreased the TRAP‐positive cells and lipid droplets in osteoclastogenic culture of mBMDMs at day 4 (*n* = 3–4 per group; Figures 7d,e,f,g). When GSK2033, a lipid accumulation activator,^[^ ^52^ ^]^ was introduced into osteoclastogenic culture of mBMDMs, TRAP‐positive cells and lipid droplets were significantly increased at day 4 (*n* = 3‐4 per group; Figures 7d,e,f,g).

To examine whether the exposure of PE on the cell surface is functionally involved in osteoclast fusion, cinnamycin which specially binds to PE,^[^ ^53^ ^]^ was introduced into osteoclastogenic culture of mBMDMs (containing both M‐CSF and RANKL). No TRAP‐positive cells was visible anymore at day 4 (*n* = 3 per group, Figure 7h,i). When CD36, a molecular mediator of macrophage fusion,^[^ ^54^ ^]^ was blocked with anti‐CD36 antibody, both the number and area of TRAP‐positive cells significantly decreased in osteoclastogenic culture of mBMDMs (*n* = 3 per group, Figure 7h,i).

### 2.8 Pharmacologic Activation of M2 Enhances Lipid Accumulation in Macrophage During Osteoclastogenesis

**Figure 8:**
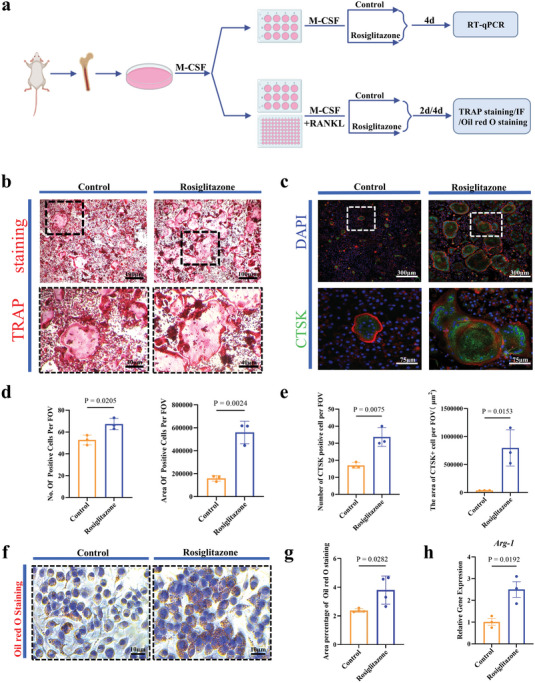
Influence of rosiglitazone on macrophage polarization and osteoclastogenesis in vitro. a) Schematic diagram of the experimental design. b) TRAP staining of osteoclasts (*n* = 3). c) CTSK immunofluorescence staining of osteoclasts (*n* = 3). d) Number and area of TRAP‐positive cells in osteoclastogenesis culture (*n* = 3). Two‐tailed Student's *t*‐test. e) Number and area of CTSK‐positive cells in osteoclastogenesis culture (*n* = 3). Two‐tailed Student's *t*‐test. f) Oil red O staining of osteoclastogenic culture of mBMDMs treated with rosiglitazone for 2 days (*n* = 4). g) Area of percentage Oil red O staining of osteoclastogenic culture of mBMDMs treated with rosiglitazone for 2 days (*n* = 4). Two‐tailed Student's *t*‐test. h) *Arg‐1* gene expression of mBMDMs cultured in M‐CSF containing medium with/without rosiglitazone for 4 days (*n* = 3). Two‐tailed Student's *t*‐test. Each error bar represents the mean ± SD. Differences were considered statistically significant at *p* < 0.05.

When mBMDMs were cultured with either M‐CSF alone or with both M‐CSF and rosiglitazone (an M2 activator^[^ ^55^ ^]^) for 4 days (*n* = 3 per case) (**Figure** **8**a), rosiglitazone increased significantly *Arg‐1* gene expression in mBMDMs culture at day 4 (Figure 8h). When rosiglitazone was introduced into osteoclastogenic medium (containing both M‐CSF and RANKL), it increased significantly osteoclasts in number, in cells size and in area in osteoclastogenic culture of mBMDMs at day 4 (*n* = 3 per case) (Figure 8b–e). At the same time, rosiglitazone significantly increased the number and area of lipid droplets in osteoclastogenic culture of mBMDMs at day 2 (*n* = 4 per case) (Figure 8f,g).

### 2.9 Condition Medium from Osteoclastogenic Culture Enhances Osteogenic Differentiation of MSCs

**Figure 9:**
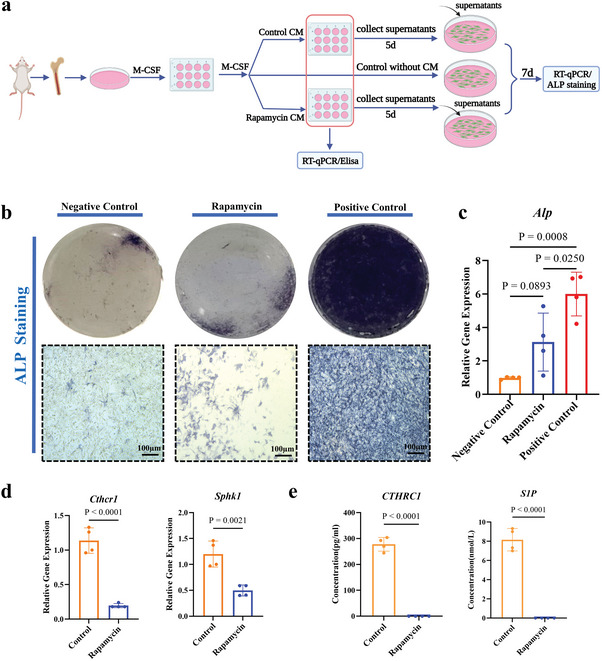
Osteogenic function of osteoclastogenesis. a) Schematic diagram of the experimental design. b) ALP staining of the MSCs after cultured in different media for 7 days (*n* = 4). c) *Alp* gene expression of mBMSCs after cultured in different media for 7 days (*n* = 4). One‐way ANOVA with Tukey's post‐test. Negative control: MSCs were cultured with basic media; Rapamycin: MSCs were cultured with conditioned media (osteoclast media were collected after osteoclastogenesis induction with rapamycin treatment for 5 days); Positive control: MSCs were cultured with conditioned media (osteoclast media were collected after osteoclastogenesis induction for 5 days). d) *Cthrc1* and *Sphk1* gene expression of mBMDMs cultured with osteoclastogenic medium (containing both M‐CSF and RANKL) plus and minus rapamycin for 5 days (*n* = 4). Two‐tailed Student's *t*‐test. e) The level of CTHRC1 and S1P proteins secreted by mBMDMs cultured with osteoclastogenic medium (containing both M‐CSF and RANKL) plus and minus rapamycin for 5 days (*n* = 4). Two‐tailed Student's *t*‐test. Each error bar represents the mean ± SD. Differences were considered statistically significant at *p* < 0.05.

The influence of osteoclastogenesis on osteogenic differentiation of MSCs was evaluated by culturing MSCs with/without osteoclastogenesis condition medium and condition medium with the presence of rapamycin for 7 days (*n* = 4 per case) (**Figure** **9**a). The influence of rapamycin on osteogenic differentiation of MSCs was evaluated by culturing MSCs with osteogenic medium with/without rapamycin for 7 days (*n* = 4 per case) (Figure S5a, Supporting Information), it turned out that at the concentration same as to that in condition medium in the test group (osteoclastogenesis condition medium plus rapamycin), rapamycin did not inhibit osteogenic differentiation of MSCs (Figure S5a,b, Supporting Information). Alkaline phosphatase (ALP) staining of MSCs culture at day 7 showed that osteoclastogenesis condition medium (positive control) enhanced ALP production as compared to the negative control (without osteoclastogenesis condition medium), while such an osteogenic function of osteoclastogenesis condition medium decreased when rapamycin was introduced into the osteoclastogenic culture of mBMDMs (Figure 9b). In addition, *Alp* gene expressed in the same way as ALP production (Figure 9c). At the same time, in the in vitro osteoclastogenesis evaluation system, rapamycin sharply down regulated both *Cthrc1* gene and *Sphk1* gene expression at day 5 (Figure 9d) and decreased the level of CTHRC1 and S1P proteins secreted by mBMDMs cultured with M‐CSF and RANKL for 5 days (Figure 9e).

## 3 Discussion

When TCPB and MG were implanted in muscles of FVB mice, newly formed bone was histologically detected in MG at week 6, no bone was observed in TCPB (Figure 1b,c,e). The results are in agreement with the previous data obtained in a canine intramuscular implantation model,^[^ ^46^ ^]^ confirmed the material‐dependent nature of material‐induced heterotopic bone formation^[^ ^13^ ^]^ and verified gluteal muscular implantation of FVB mice as a valid in vivo model to study material‐induced heterotopic bone formation.

Cells in both TCPB and MG implants became more hypoxic from week 1 to week 2, with MG implants more hypoxic than TCPB implants (Figure 4b). One reason for the difference of hypoxia in MG and TCPB implants may be related to more neutrophils recruited to osteoinductive ceramics when compared with non‐osteoinductive ceramics. It was reported that “infiltrating neutrophils at sites of inflammation contribute to hypoxia, as these cells require high levels of oxygen to support the oxidative burst” in the literature.^[^ ^56^ ^]^ However, how the materials affect neutrophils is unknown and worthy to be studied further. Another reason for the different hypoxic status in material implants could be the influence of materials on macrophage proliferation, resulting the different numbers of macrophages in a given space. As shown in previous studies, osteoinductive materials enhance macrophage proliferation in vitro,^[^ ^40^ ^]^ and more M2 macrophages could be detected in osteoinductive material implants in vivo.^[^ ^39^ ^]^ The up‐regulated hypoxia‐related genes in MG implants (Figure 1i) confirmed an involvement of hypoxia in material‐induced heterotopic bone formation. The role of hypoxia in material‐induced heterotopic bone formation was further confirmed by the activation and inhibition of HIF‐1*α*, a master regulator of cellular responses to hypoxia.^[^ ^57^ ^]^ With higher *Hif‐1α* gene expressions at week 1 and week 2 compared to TCPB implants (Figures 1j and 4b), heterotopic bone was formed in MG implants at week 6 (Figure 1b,c,e). Moreover, inhibition of HIF‐1*α* with rapamycin and PX‐478 within 2 weeks after surgical implantation inhibited material‐induced bone formation in MG implants (Figure 4d–f; Figure S4b–d, Supporting Information). Of note, the difference in gene expression of *Hif‐1α* was disappeared at week 3 and 4, and application of rapamycin at week 3 to week 4 or week 5 to week 6 did not inhibit bone formation in MG at week 6 (Figure 4b,d–f). Furthermore, enhancing HIF‐1*α* in 2 weeks after surgical implantation with DFO enhanced material‐induced heterotopic bone formation in MG implants (Figure S4b–d, Supporting Information). More importantly, enhancing HIF‐1*α* in 2 weeks after surgical implantation with DFO made it possible even for non‐osteoinductive TCPB to give rise to heterotopic bone formation (Figure S4b–d, Supporting Information). The role of hypoxia in material‐induced heterotopic bone formation as demonstrated herein explained well in one hand the geometrical control of material‐induced heterotopic bone formation (the material‐induced bone formation occurs in 3D macroporous environment created by either concave surface or macropores^[^ ^13^, ^58^, ^59^, ^60^ ^]^ and made sense that material‐induced bone formation starts inside the implants,^[^ ^13^, ^39^, ^58^, ^59^, ^60^, ^61^ ^]^ on the other hand, since a hypoxic status could be easily reached in concave surface and inside the implants).

Difference of angiogenesis between TCPB implants and MG implants at week 1 and week 2 was not seen with neither gross observation of CD31, *α*‐SMA, and EMCN immunostainings (Figure S2b, Supporting Information), nor transcriptomic analysis for angiogenesis‐related gene expression (Figure S2c, Supporting Information), nor RT‐qPCR for gene expression of *Cd31*, *Emcn*, and *Vegfa* (Figure S2d, Supporting Information), indicating that the role of angiogenesis in material‐induced heterotopic bone formation is limited and angiogenesis may not be the mechanism by which hypoxia play its role in HO.

Upregulation of M2 marker gene and strong staining of M2 macrophage marker (e.g., ARG‐1) was observed in osteoinductive implants compared to non‐osteoinductive implants at week 1 (Figure 2c,f), with no significant difference of M1 macrophage marker (e.g., CCR7) (Figure S3a, Supporting Information), indicating the roles of M2 macrophages in material‐induced bone formation as previously reported.^[^ ^35^, ^36^, ^37^ ^]^ The up‐regulation of *Hif‐1α* gene and stronger HIF‐1*α* immunostaining (Figure 2b,e) accompanying macrophage polarization to M2 indicated that hypoxia play its role in material‐induced bone formation via macrophage polarization. Meanwhile, both HIF‐1*α* activation and M2 macrophage polarization were negatively regulated by HIF‐1*α* inhibitors (e.g., Rapamycin and PX‐478) (Figure 2). Moreover, the matching of HIF‐1*α* staining and ARG‐1 staining in MG implants at week 1 (Figure 2d) and the co‐existence of CD206‐positive cells and pimonidazole‐positive cells in MG implants at week 1 (Figure 1g) in double immunofluorescence staining suggested that M2 macrophages were enriched responding to hypoxia. Furthermore, in agreement with literatures,^[^ ^62^, ^63^, ^64^ ^]^ low oxygen enhanced M2 macrophages in vitro while rapamycin inhibited M2 macrophages (Figure 5b,c,e). The data supported the M2 macrophage mechanism by which hypoxia play its role in HO.

In line with previous finding that osteoclasts formed in osteoinductive materials after macrophage polarization to M2 (e.g., week 1) but prior to the occurrence of bone (e.g., after week 4),^[^ ^39^ ^]^ osteoclasts were observed in MG implants at week 2 (Figure 3d–g), confirming the role of osteoclastogenesis in material‐induced bone formation.^[^ ^38^, ^39^, ^40^, ^41^, ^42^ ^]^ Since MG implants became more hypoxic and HIF‐1*α* up‐regulation at the same time at week 2 (Figure 1), it is likely that hypoxia play its role through osteoclastogenesis. Such a possibility was further confirmed by both in vivo and in vitro data. When rapamycin and PX‐478 (both are HIF‐1*α* inhibitors) were applied intraperitoneally in the first 2 weeks after surgery, HIF‐1*α* was down regulated in MG implants within 2 weeks (Figure 3b,c), osteoclastogenesis was inhibited in MG implants at week 2 (Figure 3d–g) and bone formation in MG implants inhibited accordingly at week 6 (Figure 4). Moreover, when rapamycin was introduced into the in vitro osteoclastogenesis evaluation system, formation of osteoclasts was strongly decreased (Figure 5d,f–h; Figure S7, Supporting Information). Furthermore, osteoclastogenesis was significantly enhanced when low oxygen was applied to the culture (Figure 5d,f–i).

Although angiogenesis is an important process in bone formation in general,^[^ ^65^ ^]^ the role of angiogenesis in initiating material‐induced heterotopic bone formation was not found in the current study. The current data indicated macrophages (esp. M2 macrophages) and osteoclasts as the mediators between hypoxia and HO.

Hypoxia plays roles in HO,^[^ ^25^ ^]^ while it may not be able to initiate itself heterotopic bone formation, otherwise HO should happen in high rates since hypoxia microenvironments could be reached in many cases.^[^ ^66^, ^67^ ^]^ Hypoxia enhances macrophage polarization toward M2 macrophages and it was thought that M2 macrophages mediated the influence of hypoxia on bone formation (incl. HO).^[^ ^23^, ^25^ ^]^ However, M2 macrophage may not be the real mediator between hypoxia and HO. Next to their possible roles in bone formation,^[^ ^23^, ^68^ ^]^ M2 macrophages are responsible for fibrosis by producing pro‐fibrotic cytokines/growth factors.^[^ ^69^, ^70^ ^]^ A driving force is needed in determining the fate of M2 macrophages to favor fibrosis or bone formation. Osteoclastogenesis following M2 macrophages could be such the driving force. As shown in the current study and previous studies, osteoclasts were often seen prior to material‐induced bone formation and the occurrence of material‐induced bone formation was always associated with the formation of osteoclasts. Not only in material‐induced bone formation, osteoclasts played roles in ectopic bone formation in tissue‐engineered bone graft. Ectopic bone formation MSC‐based CaP biomaterials was significantly inhibited by anti‐RANKL antibody.^[^ ^44^ ^]^ Moreover, osteoclastogenesis occurred after macrophage polarization and M2 macrophages favored osteoclastogenesis,^[^ ^45^, ^71^ ^]^ It is most likely that M2 macrophages play their roles in bone formation (including heterotopic bone formation) via osteoclasts.

Osteoclastogenesis may be the real mediator between hypoxia and HO. It appeared that via the axis of M2/lipid‐loaded macrophages, hypoxia enhances osteoclastogenesis. More than just promoting macrophage polarization toward M2, hypoxia enhanced lipid accumulation in macrophages.^[^ ^72^, ^73^ ^]^ The link between hypoxia, lipid metabolism and osteoclast formation were shown in this study. As shown in Figure 6, adding HIF‐1 inhibitor (e.g., rapamycin) in culture of mBMDMs resulted in down regulation of lipid accumulation‐related genes and decrease of total lipid production of the cells (Figure 6d,g), and finally the decrease of osteoclast formation (Figure 5d,f–h; Figure S7, Supporting Information). In contrast, low oxygen significantly enhanced lipid accumulation in macrophages, and finally increased osteoclastogenesis. The importance of lipid accumulation in osteoclastogenesis was further confirmed when GW3965 and GSK2033 were introduced into the in vitro osteoclastogenesis evaluation system. As the agonist of LXR which is crucial in lipid metabolism, GW3965 decreased the lipid accumulation in macrophages (Figure 7d,g) and the formation of osteoclasts (Figure 7e,f); while as the antagonist, GSK2033 increased lipid accumulation in macrophages (Figure 7d,g) and the formation of osteoclasts (Figure 7e,f). More interestingly, it has been demonstrated that M2 macrophages are more prone to form foam cells.^[^ ^73^, ^74^ ^]^ Increasing of intracellular lipids and osteoclast formation in osteoclastogenic culture of mBMDMs with rosiglitazone further confirmed the involvement of an axis of M2/lipid‐loaded macrophages in the formation of osteoclasts (Figure 8). Among all lipids, PE and may be responsible for osteoclast fusion,^[^ ^75^ ^]^ with an obvious decrease of PC and PE when osteoclastogenesis was inhibited by rapamycin (Figure 6f). The critical role of PE in osteoclastogenesis was shown by inactivating PE with cinnamycin in osteoclastogenic culture of mBMDMs (Figure 7i,h). Specific macrophage scavenger receptors responsible for macrophage fusion by recognizing and uptaking modified cholesterol [such as oxidized low density lipoprotein (oxLDL)] to form lipid‐loaded macrophages, especially CD36,^[^ ^54^ ^]^ showed their roles in osteoclastogenesis. Blocking CD36 with anti‐CD36 antibody prevented osteoclast formation in osteoclastogenic culture of mBMDMs (Figure 7h,i). It is likely that lipid loading switches M2 macrophages to form osteoclasts instead of producing fibrogenic factors and via affecting macrophage scavenger receptors (e.g., CD36) for lipid loading and influencing PE for macrophage fusion, hypoxia effects osteoclastogenesis.

Finally, the cytokines produced in osteoclastogenesis could initiate osteogenic differentiation of MSCs to form bone and heterotopic bone formation occurs. Condition medium prepared with supernatant harvested from normal osteoclastogenesis culture at day 4 enhanced both *Alp* gene expression (Figure 9c) and ALP production (Figure 9b) of MSCs cultured for 7 days as compared to the bare control without supernatant in osteoclastogenesis culture, while supernatant of osteoclastogenesis culture with HIF‐1*α* inhibitor (e.g., rapamycin) inhibited both *Alp* gene expression and ALP production of MSCs (Figure 9b,c). Although the osteogenic factors secreted by osteoclasts are not fully known yet,^[^ ^39^, ^45^ ^]^ the expression of Cthrc1 and Sphk1 genes during osteoclastogenesis in vitro was significantly down‐regulated and the protein levels of CTHRC1 and S1P in the supernatant were significantly decreased with rampamycin (Figure 9d,e), indicating that in addition to others like BMP6, Wnt10b, C3a,^[^ ^45^, ^76^ ^]^ CTHRC1 and S1P, are responsible for the osteogenic function of osteoclasts.

The data presented herein showed clearly the involvement of hypoxia in heterotopic bone formation. While against the previous suggestion,^[^ ^25^ ^]^ hypoxia does not play its roles in heterotopic bone formation via angiogenesis. In agree with the previous findings,^[^ ^23^ ^]^ inflammation plays roles in heterotopic bone formation. Such the roles could be seen in the influence of intraperitoneal injection and peri‐implant injection of PBS on material‐indued bone formation, with enhanced bone formation in MG implants following peri‐implant injection of PBS (Figure 1e; Figure S4c,d,e, Supporting Information), because of the local inflammation caused by injury. In line with the previous hypothesis,^[^ ^23^, ^25^ ^]^ M2 macrophages play roles in heterotopic bone formation. However, M2 macrophages may not be capable to initiate directly heterotopic bone formation, lipid loading followed by fusion to osteoclasts is necessary for M2 macrophages to accomplish their missions in heterotopic bone formation.

Although the process how osteoclasts promote heterotopic bone formation needs to be further addressed, these findings indicated the pro‐osteogenic function of osteoclasts in HO and bone regeneration in osseous defects^[^ ^45^, ^77^ ^]^ and thus point toward a potential novel approach for preventing pathological ossification and improving bone regeneration by manipulating osteoclastogenesis. Given the fact that heterotopic bone formation (bone formation far away from the host bone bed) is an advantage of osteoinductive materials and osteoclastogenesis is the driving force of material‐induced bone formation, this finding may imply that elderly patients and osteoporotic patients could benefit more from osteoinductive materials for bone repair because their overall condition favors osteoclastogenesis. Moreover, these findings could be helpful in designing novel bone substitutes. Physicochemical modification of materials to polarize macrophages to M2 macrophages and subsequent osteoclastogenesis could improve the bone‐forming potentials of osteoinductive materials. Interestingly, the involvement of hypoxia and no apparent influence of angiogenesis on material‐induced bone formation observed in this study do not support the design principle of enhancing angiogenesis in bone grafting materials.^[^ ^78^ ^]^ Lastly, material‐induced bone formation can serve as a useful model to study bone biology (e.g., the role of osteoclasts), to evaluate the mechanism of HO and treatment of diseases relevant to HO.

## 4 Conclusion

**Scheme 1:**
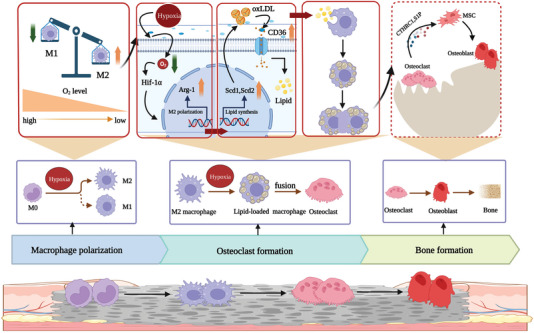
Via the axis of M2/lipid‐loaded macrophages, hypoxia enhances osteoclastogenesis, thus promotes material‐induced heterotopic bone formation.

The overall data demonstrated a strong link between hypoxia, macrophage polarization to M2, osteoclastogenesis, and material‐induced bone formation. Hypoxia enhances macrophage polarization to M2, stimulates lipid loading into M2 macrophage to facilitate osteoclastogenesis, factors generated in osteoclastogenesis initiate heterotopic bone formation (**Scheme** **1**). These findings pin‐point a mechanism to understand heterotopic bone formation, pave a novel way for the design of bone grafting materials and provide strategies for the prevention and treatment of HO.

## 5 Experimental Section

### Materials

Porous discs of TCPB and MG (Φ5.0 × 2.5 mm) were provided by Kuros Biosciences BV, the Netherlands. Preparation of the materials was reported previously^[^ ^44^ ^]^ and the physicochemical properties of the materials were illustrated as Supporting Information (Figure S1, Supporting Information). The materials were sterilized at 160 °C for 120 min before uses.

### Animals

FVB mice (male, 6–7 weeks old, Charles River Laboratory, China) were used in this study. Mice were raised in temperature‐controlled environment with artificial 12 h light/dark cycles and were fed with normal diet. Animal experiments were conducted following the ARRIVE guidelines and approved by the Animal Care and the Ethics Committee of Stomatological Hospital of Chongqing Medical University (2022(098)). Animals were randomly assigned to the controls or treatment groups in a blinded fashion.

### Surgical Implantation

The intramuscular implantation model was applied in this study. Mice were subjected to inhalation of 2% isoflurane (ISOTHESIA, UK) for anesthesia and subcutaneous injection of buprenorphine (0.1 mg kg^−1^, Buprenex Injection, USA) for analgesia. The operative area was shaved and then sterilized with iodophors and 75% ethanol respectively. Gluteal muscle pouches were created at both sides and materials (Φ5.0 × 2.5 mm) were implanted into the muscle pockets. If both TCPB and MG were compared, TCPB and MG were implanted in the same animals randomly on left side or right side; Otherwise, materials were implanted if were implanted unilaterally in a random manner (either left side or right side). The wounds were sutured with 5.0 tension‐free sutures.

### Pharmacological Treatments

To investigate the effect of HIF‐1*α* inhibitors/activators on bone formation in vivo, mice were administered rapamycin (5 mg kg^−1^, AbMole, USA), PX‐478(50 mg kg^−1^, MCE, USA) or DFO (4 mg kg^−1^, AbMole, USA) via intraperitoneal injection or peri‐implant injection. Rapamycin and PX‐478 were dissolved in solvent solution containing 0.2% carboxymethylcellulose and 0.25% Tween‐80 in sterile H_2_O. DFO was dissolved in PBS. Mice received intraperitoneal injection or peri‐implant injection every other day between 0–2 weeks, 2–4 weeks or 4–6 weeks according to experimental design. PBS injection alone was used as the control group if necessary.

### Sample Harvesting

At pre‐determined time points, the mice were sacrificed by cervical dislocation after a general anesthesia with carbon dioxide. The implants were carefully separated from the surrounding tissues and stored in either a 1.5 mL Eppendorf (EP) tube containing RNALater Animal Tissue RNA Stable Preservation Solution (Beyotime, China) for RNA isolation or fixed in 4% paraformaldehyde (Servicebio, China) for histological and immunohistochemical analyses.

### Histology

For non‐decalcified sections, the samples were dehydrated in ethanol with gradient concentrations and embedded in polymethyl methacrylate (CoolSet A, Aorigin, Chengdu, China). Non‐decalcified sections (10–20 µm) were obtained with a diamond histological saw (SAT‐001, Aorigin, Chengdu, China) and stained with 1% methylene blue (Sigma‐Aldrich, Germany) and 0.3% basic fuchsin (Sigma‐Aldrich, Germany) solutions. For decalcified sections, explants were decalcified with EDTA decalcification solution (slow decalcification) (ServiceBio, China), dehydrated with a series ethanol solution, and embedded in paraffin (Leica, Germany) to get paraffin sections (4–7 µm) for Masson's trichrome staining, TRAP staining, and immunohistochemical and immunofluorescence staining.

### Masson's Trichrome Staining

Decalcified paraffin sections were first deparaffinized with xylene and ethanol, then stained with a Masson's trichrome staining kit (Solarbio, China) following the instruction for use. Sections were mounted with neutral balsam.

### TRAP Staining

Decalcified paraffin sections were first deparaffinized with xylene and ethanol, incubated with pure water at 37 °C for 2 h, TRAP staining was performed using a commercial TRAP staining kit (Servicebio, China) at 37 °C to avoid light reaction at 20–30 min according to the instruction. Sections were counterstained with hematoxylin (Solarbio, China) thereafter and mounted with neutral balsam. Cells were fixed in 4% paraformaldehyde for 30 min, then treated with 0.3% Triton permeabilization solution for 30 min, and stained with a TRAP staining kit (ServiceBio, China) following the instruction for use. Images were taken under the microscope at 4× and 10× and analyzed with Image‐Pro Plus 6.0 software to get both TRAP‐positive cell numbers and TRAP‐positive area per view of field at 4×.

### Immunohistochemical Staining

Decalcified paraffin sections were first deparaffinized with xylene and ethanol, pre‐treated with an immunohistochemistry detection system kit (Bioss, China) and subjected to immunohistochemical staining. Briefly, sections were incubated at 4 °C overnight with primary antibodies (HIF‐1*α* (1:100, HUABIO, China), ARG‐1 (1:100, ABclonal, China), CCR7 (1:200, Servicebio, China), F4/80 (1:500, Servicebio, China), and CTSK (1:200, Proteintech, China)), incubated subsequently with secondary antibody and finally visualized with DAB chromogen (Beyotime, China), counterstained with hematoxylin (Solarbio, China), and mounted with neutral balsam.

### Immunofluorescence Staining

The immunofluorescence staining procedure in the first day was the same as those for immunohistochemical staining, the only difference was without inactivation of endogenous peroxidase. The primary antibodies included HIF‐1*α* (1:100, HUABIO, China), ARG‐1 (1:100, ABclonal, China), CD206 (1 µg mL^−1^, Abcam, UK), CCR7 (1:200, Servicebio, China), CD31 (1:200, HUABIO, China), *α*‐SMA (1:200, HUABIO, China), and EMCN (1 µg mL^−1^, Abcam, UK), as well as the secondary antibodies involved goat anti‐rabbit IgG H&L Alexa Flour 488 (1:200, Abcam, UK), goat anti‐rabbit IgG H&L Alexa Flour 555 (1:200, Abcam, UK) and goat anti‐rabbit IgG H&L Alexa Flour 647 (1:200, Abcam, UK). DAPI stain was applied to visualize nuclei (Beyotime, China). Images were captured under a fluorescent microscope (Leica, Germany).

### Hypoxia Staining

Hypoxia staining was determined using a Hypoxyprobe Plus Kit (Hypoxyprobe, USA). Mice were injected intraperitoneally with pimonidazole (60 mg kg^−1^, Hypoxyprobe, USA) dissolved in H_2_O one hour prior to sample harvesting. The samples directly frozen in liquid nitrogen and were embedded with O.C.T. compound (Sakura, USA) to get 5 µm consecutive sections. The sections were fixed in cold 4% paraformaldehyde (Servicebio, China) for 10 min, merged into the sodium citrate buffer for 10 min under 95 °C for antigenicity restore and treated with 3% H_2_O_2_ to inactivation of endogenous peroxidase. After blocked with 5% goat serum (diluted in PBS, Beyotime, China) at room temperature for 60 min. Sections were then incubated with the FITC conjugated to anti‐pimonidazole mouse IgG1 monoclonal antibody (1:100, Hypoxyprobe, USA) at 4 °C overnight. At the second day, nucleus was visualized with DAPI (Beyotime, China). Images were captured under a fluorescent microscope (Leica, Germany).

### Histomorphometry

Quantification of TRAP staining, immunohistochemical staining, and immunofluorescence staining was performed with decalcified sections. Briefly, the stained decalcified sections were digitally scanned (Olympus, Japan) to images for analysis, positive cells per field of view (FOV) with the view size of 1438 µm × 1204 µm in the representative sections were quantified using Image‐Pro Plus 6.0 software. Quantification of bone was conducted with all non‐decalcified sections obtained from the entire explant per sample. In short, the sections were digitalized with a scanning microscope (Aimicro, Xiamen, China) to get overviews, and percentage of bone in available space was quantified (Area of bone × 100/area of region of interest‐area, i.e., the area of CaP ceramic implants) with Image‐Pro Plus 6.0 software.

### RT‐qPCR

Total RNA of animal and cell samples was isolated by RNA extraction kit (Beyotime, China), and cDNA was then acquired using PrimeScript RT Master Mix (TAKARA, Japan). Using TB Green Premix Ex Taq II (TAKARA, Japan) as the reaction solution, Real‐time PCR reaction was proceeded at 95 °C for 3 min, followed by 95 °C for 10 s, 60 °C for 1 min for 40 cycles. Gene expressions were then measured using the ABI StepOnePlus Real‐Time PCR System. *Gapdh* was chosen as the housekeeping gene for normalization. *Hif‐1α*, macrophage polarization‐related genes [M2: *Arg‐1*, *Cd163*], osteoclast‐related genes [*Trap*, *Ctsk*], and angiogenesis‐related genes [*Vegf*, *Cd31*, *Emcn*] were targeted and their primer sequences were listed in Table S1, Supporting Information. 2^−△△Ct^ method was used to quantitively analyze the relative gene expression and GraphPad Prism 9.4 software (GraphPad Software, USA).

### Transcriptome Sequencing and Data Analysis

The explants of TCPB and MG were harvested at week 1 and week 2 for transcriptome analysis. Briefly, explants were lysed with the Invitrogen TRIzol Reagent to extract cDNA. The cDNA library was constructed using the SuperScript double‐stranded cDNA synthesis kit (Invitrogen, CA). The Illumina HiSeq TM2500 sequencing platform (SeqHealth Tech Co., Ltd., Wuhan, China) was used for high‐throughput sequencing.

### Culture of mBMDMs and mBMSCs

The tibiae of male mice (6–7 weeks old) were aseptically collected, and the marrow were flushed out with basic medium (1640 medium containing 10% fetal bovine serum (ExCellent Bio, Australia) and 1% v/v penicillin/streptomycin (HyClone, USA)). The marrow suspension was subsequently sieved with a 40 µm cell strainer and treated with lysate (Beyotime, China), and subjected to culture with the basic medium with the presence of 70 ng mL^−1^ M‐CSF (Sino Biological, China). Non‐adherent cells were collected 24 h later and cultured for 3 days to get the adherent cells ready as mBMDMs. The cells were harvested with a cell scraper for further use.

CRL‐12424 cells (a mesenchymal stem cell line derived from mouse bone marrow) were purchased from ATCC and maintained in basic *α*‐MEM medium consisting of *α*‐modified eagle medium (*α*‐MEM, SIGMA, UK) supplemented with 10% fetal bovine serum and 1% v/v penicillin/streptomycin. The cells were harvested with 0.25% trypsin/EDTA (SIGMA, UK) for further use.

### Macrophage Polarization of mBMDMs with Different Treatments

The mBMDMs were incubated in 12‐well plates for immunofluorescence staining and RT‐qPCR at a density of 2 × 10^5^ cells cm^−2^ in basic medium plus 20 ng mL^−1^ M‐CSF to analyze macrophage polarization. In the control group, cells were incubated in normal atmosphere (20% O_2_, 5% CO_2_, and 75% N_2_). For hypoxia treatment, mBMDMs were incubated in a hypoxic incubator (Thermo scientific; Asheville, NC, USA) with low oxygen tension (6% O_2_, 5% CO_2_, and 89% N_2_). For rapamycin treatment, 0.5 µm rapamycin (MCE, USA) was introduced in the culture medium and culture in normal atmosphere. The media were refreshed every other day.

### Osteoclastogenesis of mBMDMs with Different Treatments

For immunofluorescence staining and RT‐qPCR, mBMDMs were incubated in 12‐well plates. For TRAP staining, mBMDMs were incubated in 96‐well plates. A seeding density of 2 × 10^5^ cells cm^−2^ was used. Osteoclastogenesis induction was started at day 0 with medium containing 100 ng mL^−1^ M‐CSF (Sino Biological, China) and 100 ng mL^−1^ RANKL (PeproTech, USA). Medium was changed every other day for 5 days. Rapamycin and hypoxia treatments were the same as described in Section 5.4.2. In the case of other chemical treatments, chemicals (Rosiglitazone, (AbMole, USA), 1 µm; GSK2033, (AbMole, USA), 2 µm; GW3965, (AbMole, USA), 10 µm; Cinnamycin, (ChemeGen, China), 100 µg mL^−1^; Anti‐CD36 antibody, (HUABIO, China), 10 µg mL^−1^; oxLDL, (Yiyuan Biotechnology, China), 50 µg mL^−1^) were introduced into the osteoclastogenic medium.

### Culture of CRL‐12424 Cells with Different Treatments

The CRL‐12424 cells were inoculated in 12‐well plates at a density of 1 × 10^4^ cells cm^−2^. In the case of conditioned media treatment, cells were cultured with the mixture of osteoclast media collected after osteoclastogenesis induction with/without rapamycin treatment for 5 days and basic *α*‐MEM medium (1:2 v/v ratio), with basic *α*‐MEM medium was used as the negative control. In case of the influence of rapamycin on osteogenesis of MSCs, CRL‐12424 cells were cultured with osteogenic medium [basic *α*‐MEM medium containing 10 mm *β*‐glycerophosphate (*β*‐GP) (Beyotime, China), 1.0 × 10^−8^ m dexamethasone (Dex) (Beyotime, China), and 50 µg mL^−1^ vitamin C (VitC) (Beyotime, China) plus/minus rapamycin (0.17 µm). The medium was changed every other day. Samples were collected after 7 days to evaluate osteogenic differentiation of CRL‐12424 cells.

### Immunocytochemistry

Cells were fixed with 4% paraformaldehyde for 30 min and treated with 0.3% Triton X‐100 for 30 min. Non‐specific antigens were blocked with 5% donkey serum (diluted in PBS, Solarbio, China) at room temperature for 60 min, the samples were then incubated with the primary antibody (CD206, Abcam, UK, 1 µg mL^−1^; CTSK, Proteintech, China, 1:200) at 4 °C overnight and with the fluorescent secondary antibody (donkey anti‐rabbit IgG H+L, Proteintech, China, 1:100) for 1 h. Nuclei were stained with DAPI (Beyotime, China) for 10 min. Images were taken under a fluorescence microscope at 4× and 10× and analyzed with Image‐Pro Plus 6.0 software to quantify CD206‐positive and CTSK‐positive cells per FOV at 4×.

### Flow Cytometry

Cell suspensions containing approximately 1 × 10^6^ cells was centrifuged at 400 × *g* for 5 min, resuspended in 100 µL FACS (PBS with 2% FBS) and incubated with 2 µL additional Fc blocking agent (BD Biosciences, USA) at 4 °C for 5 min. After a further incubation with CD206 antibody (ThermoFisher, USA) and CD61 antibody (a marker of osteoclasts) (ThermoFisher, USA) on ice for 20–30 min, samples were then centrifuged (400 × *g* for 5 min) and resuspended in 400 uL FACS for flow cytometry. Signals were detected with a flow cytometer (CytoFLEX, Beckman Coulter), on‐board assays were performed and the data were analyzed with FlowJo software.

### Enzyme‐Linked Immunosorbent Assay (ELlSA)

ELISA was applied to supernatants of in vitro cell. The supernatants were collected from osteoclastogenesis culture of mBMDMs with/without rapamycin treatment after 5 days, centrifuged at 2000 rpm for 20 min and kept at −80 °C before use. ELISA assays of CTHRC1 and S1P were conducted with commercial kits (Elabscience, China), following the manufacturer's instructions. 4 samples were used for each group (*n* = 4).

### ALP Staining

Cells were rinsed with PBS and fixed in 4% paraformaldehyde at 37 °C for 30 min. ALP staining was performed using a BCIP/NBT Alkaline Phosphatase Color Development Kit (Beyotime, China). Images were taken under the microscope at 10× (Leica, Germany).

### Targeted Metabolome and Transcriptome association analysis

The mBMDMs (≈1 × 10^6^ cells per sample) cultured with osteoclastogenic medium with/without rapamycin for 4 days were subjected to transcriptome sequencing and targeted metabolomic analysis. Briefly, for transcriptomic analysis, cells were lysed with the Invitrogen TRIzol Reagent to extract cDNA. The cDNA library was constructed using the SuperScript double‐stranded cDNA synthesis kit (Invitrogen, USA) protocol. Illumina NovaSeq 6000 sequencing platform (Shanghai Applied Protein Technology) was performed for transcriptomic analysis. For targeted metabolomic analysis, samples were mixed with methanol, internal lipid standards and methyl tert‐butyl ether (Aladdin, USA). The mixture was adequately vortexed, sonicated and then kept for 30 min. After that, MS‐grade water (Thermo Fisher, USA) was added, and the mixture was vortexed and centrifuged. The upper organic solvent layer was obtained and dried under nitrogen. For LC‐MS analysis, the samples were re‐dissolved in IPA/CAN (9:1, v/v) solvent and then centrifuged. The analysis was performed on a UHPLC system (Nexera LC‐30A, Shimadzu) coupled with QTRAP MS (6500+, Sciex). The analytes were separated on HILIC (Phenomenex, Luna NH2, 2.0 mm ×100 mm, 3 µm) and C18 column (Phenomenex, Kinetex C18, 2.1 × 100 mm, 2.6 µm).

### Oil red O staining

Macrophages were treated with 50 µg mL^−1^ oxLDL for 2 days. After fixation in 4% paraformaldehyde for 30 min, then treated with 0.3% Triton permeabilization solution for 30 min, and stained with an oil red O staining kit (Beyotime, China) for 30 min. Cells were then stained with hematoxylin for 5 s and rinsed in PBS. Images were taken under the microscope at 40× and analyzed with Image‐Pro Plus 6.0 software to get area of percentage Oil red O staining at 40× (Leica, Germany).

### Statistical Analysis

According to different scenarios, *t*‐test, one‐way analysis of variance (ANOVA) with Tukey's post‐test multiple comparisons, and two‐way ANOVA with Bonferroni's post‐test multiple comparisons were performed by using GraphPad Prism 9.0 software. All the data were represented by mean ± SD and *p* < 0.05 was considered as a significant difference.

## Conflict of Interest

The authors declare no conflict of interest.

## Supporting information

Supporting Information

## Data Availability

The data that support the findings of this study are available from the corresponding author upon reasonable request.
