# Supplementary material for: Hypoxia Drives Material‐Induced Heterotopic Bone Formation by Enhancing Osteoclastogenesis via M2/Lipid‐Loaded Macrophage Axis
Source: Adv Sci (Weinh). 2023 Mar 27;10(15):2207224. doi: 10.1002/advs.202207224 (PMC10214238; doi:10.1002/advs.202207224)
Supplement: Supplementary file 1 — Supporting Information [file ADVS-10-2207224-s001.pdf]

ADVANCED SCIENCE
Open Access

Supporting Information

Hypoxia Drives Material-induced Heterotopic Bone Formation by Enhancing Osteoclastogenesis via M2/lipid-loaded Macrophage Axis

Dan Li[1], Yucan Jiang[1], Ping He, Yeming Li, Yan Wu, Wei Lei, Nanxin Liu, Joost D. de Bruin, Hua Zhang, Hongmei Zhang, Ping Ji, Huipin Yuan*, Mingzheng Li**

Supporting Information

Hypoxia Drives Material-induced Heterotopic Bone Formation by Enhancing Osteoclastogenesis via M2/lipid-loaded Macrophage Axis

Dan Li[1], Yucan Jiang[1], Ping He, Yeming Li, Yan Wu, Wei Lei, Nanxin Liu, Joost D. de Bruin, Hua Zhang, Hongmei Zhang, Ping Ji, Huipin Yuan*, Mingzheng Li**

a

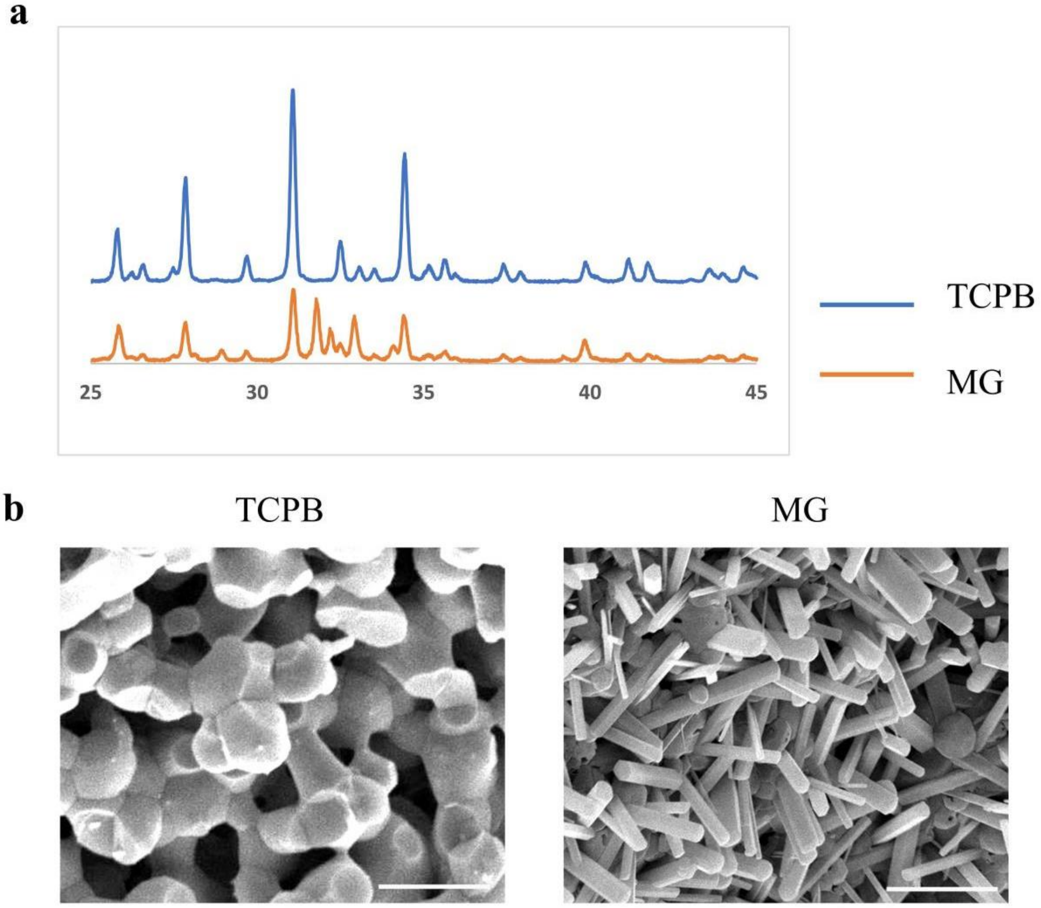

c

Material	Chemistry	Morphology of surface crystals	Scale of surface crystals (μm)
TCPB	β-TCP	Granule-like	2.40±0.54
MG	30HA/β-70TCP*	Needle-like	0.64±0.26

*By weight, HA: hydroxyapatite, TCP: tricalcium phosphate

Figure S1. Physicochemical properties of the materials. (a) X-ray diffraction of TCPB and MG; (b) Surface morphology of TCPB and MG characterized by SEM, Scale bars = 5 um; (c) A summary of physicochemical properties of TCPB and MG.

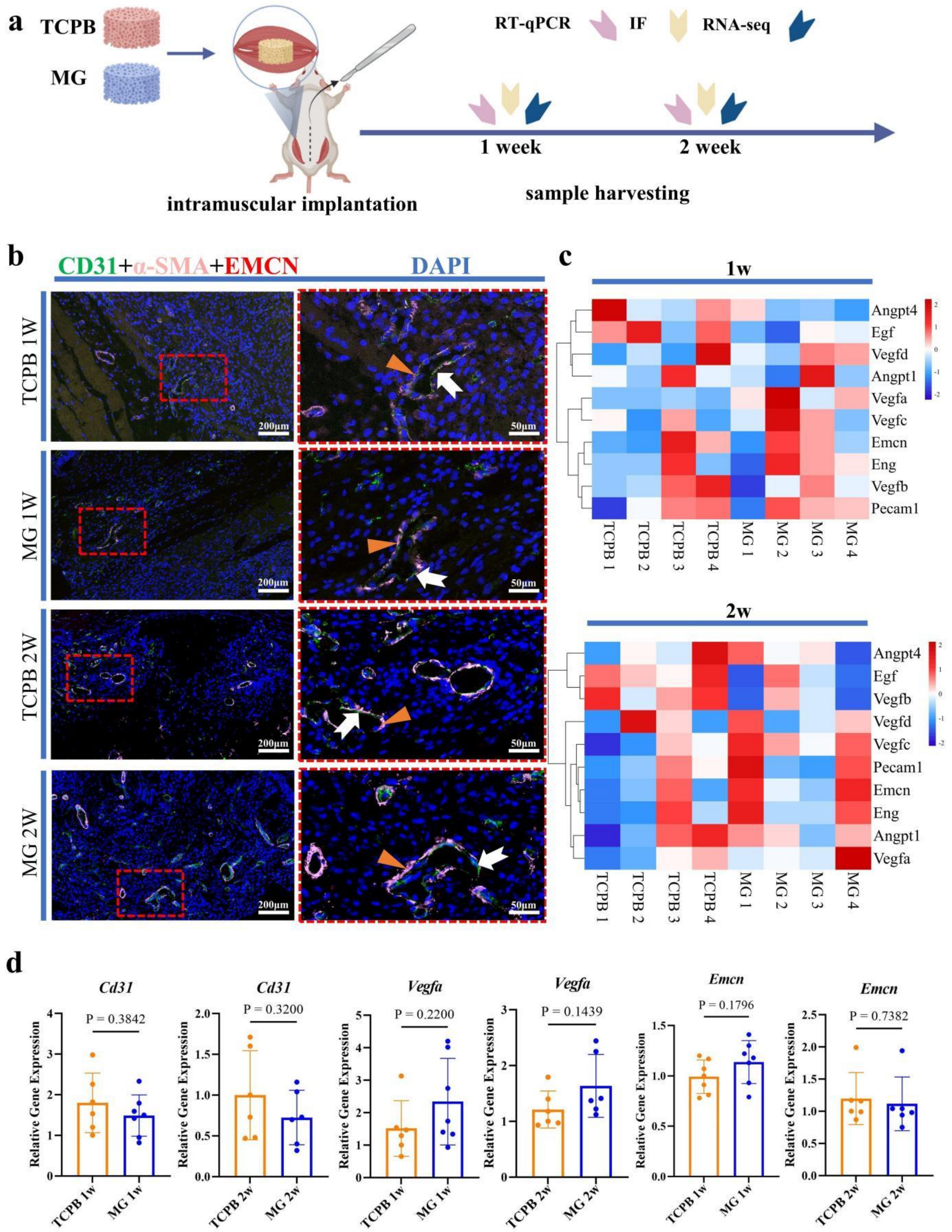

Figure S2. Angiogenesis in TCPB and MG implants at week 1 and week 2. (a) Scheme of the study design; (b) EMCN, CD31 and α-SMA multiple immunofluorescence staining (n=4); (c) Heatmap of the angiogenesis-related genes (n=4); (d) Gene expression of *Cd31*, *Emcn* and *Vegfa* (n=6-7). Two-tailed Student's t-test. The orange triangles indicate α-SMA-positive cells,

the white arrows indicate CD31-positive cells. Each error bar represents the mean ± SD. Differences were considered statistically significant at $p < 0.05$.

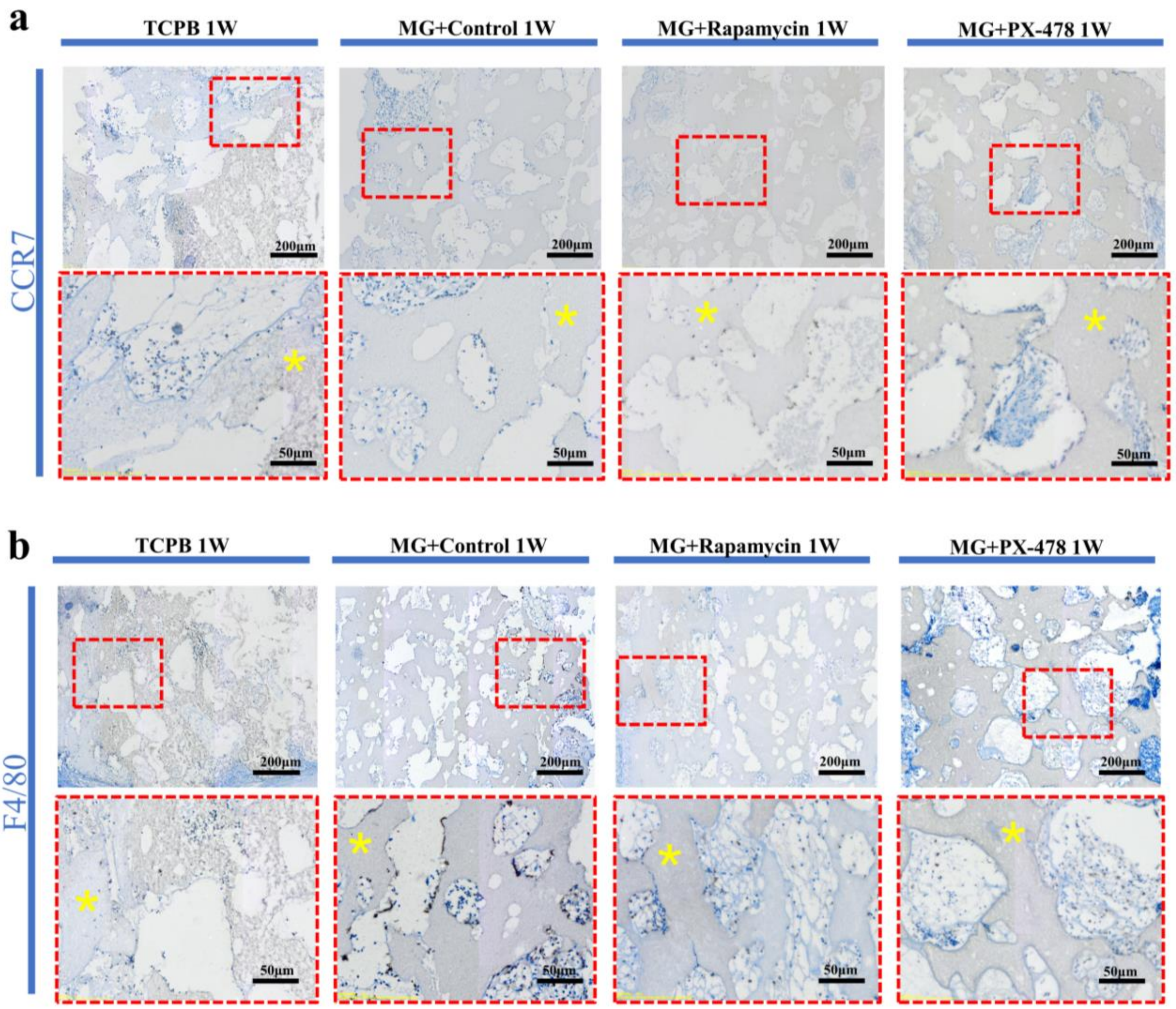

Figure S3. Influence of HIF-1α inhibitors (rapamycin and PX-478) on expression of macrophage and its polarization in MG and TCPB implants at week 1. (a) CCR7 immunohistochemical staining (n=4); (b) F4/80 immunohistochemical staining (n=4). The yellow asterisks indicate the materials.

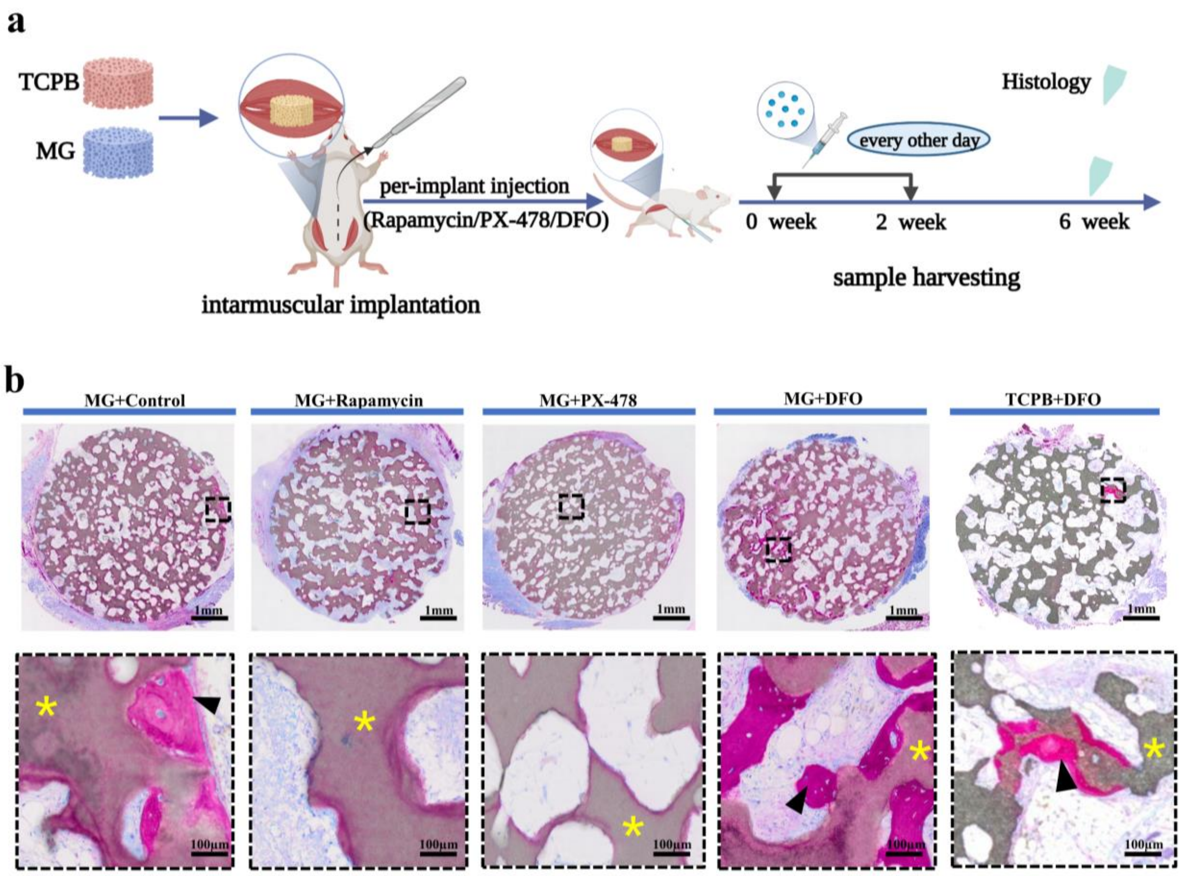

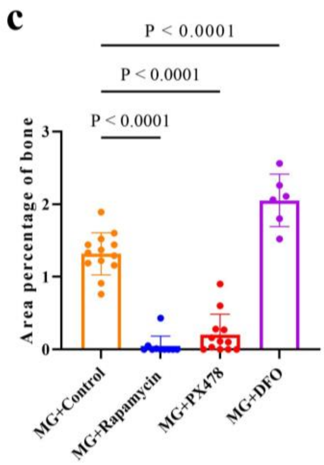

d

	MG+Control	MG+Rapamycin	MG+PX-478	MG+DFO	TCPB+DFO
Ectopic bone formation incidence rate(materials)	13/13	3/10	8/12	6/6	7/8
Ectopic bone formation incidence rate(sections)	70/82	9/63	22/70	27/27	16/50
Area percentage of bone	1.32±0.29%	0.05±0.13%	0.20±0.28%	2.05±0.36%	0.07±0.11%

e

	TCPB 6w	MG 6w
Ectopic bone formation incidence rate(materials)	0/6	6/6
Ectopic bone formation incidence rate(sections)	0/37	21/39
Area percentage of bone	0	0.76±0.33%

Figure S4. Influence of Rapamycin, PX-478 and DFO on CaP-induced bone formation following peri-implant injection for 2 weeks. (a) Scheme of the study design; (b) Histology of samples harvested at week 6 (non-decalcified sections, methylene blue/basic fuchsin stain) (n=6-

13); (c) Area percentage of bone in implants at week 6 (n=6-13). One-way ANOVA with Tukey's post-test; (d) A summary of bone formation at week 6 following peri-implant injection of chemicals (n=6-13); (e) A summary of bone formation in TCPB and MG implants at week 6 (n=6-13). The yellow asterisks indicate the materials, the black triangles indicate newly formed bone. Each error bar represents the mean ± SD. Differences were considered statistically significant at $p < 0.05$.

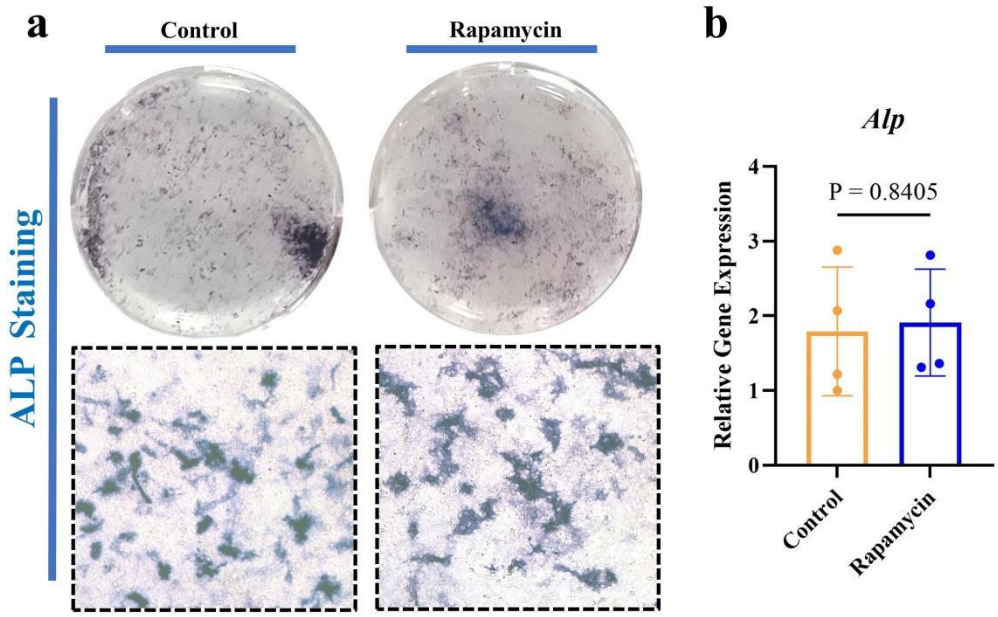

Figure S5. Osteogenic function of rapamycin. (a) ALP staining of the MSCs after cultured in osteogenic medium with/without rapamycin treatment for 7 days (n=4); (b) *Alp* gene expression of MSCs after cultured in osteogenic medium with/without rapamycin treatment for 7 days (n=4). Two-tailed Student's t-test. Control: MSCs were cultured in osteogenic medium without rapamycin treatment; Rapamycin: MSCs were cultured in osteogenic medium with rapamycin treatment. Each error bar represents the mean ± SD. Differences were considered statistically significant at $p < 0.05$.

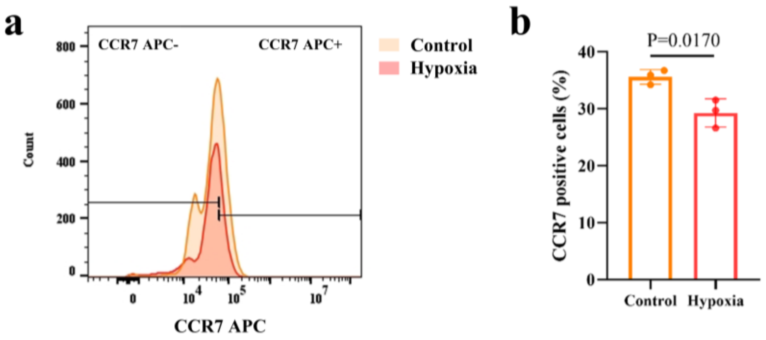

Figure S6. Influence of hypoxia on macrophage polarization of mBMDMs *in vitro*. (a) Flow cytometry for CD206 after mBMDMs cultured with M-CSF for 3 days (n=3); (b) Percentage of CD206-positive cells in mBMDMs cultured with M-CSF for 3 days (n=3). Two-tailed Student's t-test. Each error bar represents the mean ± SD. Differences were considered statistically significant at $p < 0.05$.

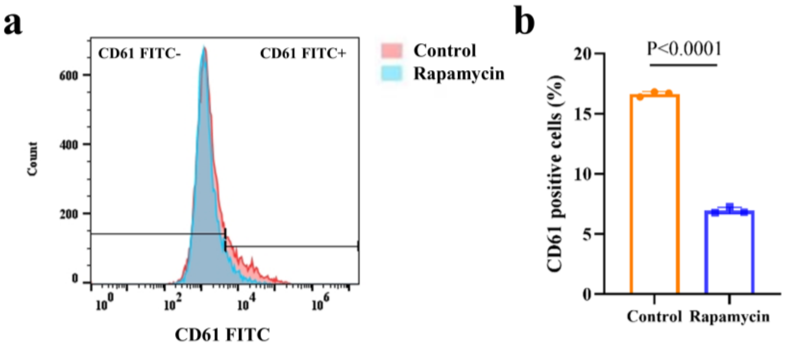

Figure S7. Influence of rapamycin on osteoclastogenesis of mBMDMs *in vitro*. (a) Flow cytometry for CD61 in mBMDMs cultured with M-CSF and RANKL for 5 days (n=3); (b) Percentage of CD61-positive cells in mBMDMs cultured with M-CSF and RANKL for 5 days (n=3). Two-tailed Student's t-test. Each error bar represents the mean ± SD. Differences were considered statistically significant at $p < 0.05$.

Table S1. Primer pairs used in the RT-qPCR

		Primer sequences (5'-3')
Hif-1α	Forward<br>Reverse	CCTGCACTGAATCAAGAGGTTGC<br>CCATCAGAAGGACTTGCTGGCT
Arg-1	Forward<br>Reverse	CATTGGCTTGCGAGACGTAGAC<br>GCTGAAGGTCTCTTCCATCACC
Cd163	Forward<br>Reverse	GGCTAGACGAAGTCATCTGCAC<br>CTTCGTTGGTCAGCCTCAGAGA
Ctsk	Forward<br>Reverse	AGCAGAACGGAGGCATTGACTC<br>CCCTCTGCATTTAGCTGCCTTTG
Trap	Forward<br>Reverse	GCGACCATTGTTAGCCACATACG<br>CGTTGATGTCGCACAGAGGGAT
Vegfa	Forward<br>Reverse	CTGCTGTAACGATGAAGCCCTG<br>GCTGTAGGAAGCTCATCTCTCC
Cd31	Forward<br>Reverse	CCAAAGCCAGTAGCATCATGGTC<br>GGATGGTGAAGTTGGCTACAGG
Emcn	Forward<br>Reverse	GCACACACCATGTCACTGCTTC<br>CAGCGCGATAACCACAGGCAAA
Alp	Forward<br>Reverse	CCAGAAAGACACCTTGACTGTGG<br>TCTTGTCCGTGTCGCTCACCAT
Cthrc1	Forward<br>Reverse	TGTTCAGGACCTCTTCCCATCG<br>GCCACATCTACCAATCCAGCAC
Sphk1	Forward<br>Reverse	GCTTCTGTGAACCACTATGCTGG<br>ACTGAGCACAGAATAGAGCCGC
Itgb3	Forward<br>Reverse	GTGAGTGCGATGACTTCTCCTG<br>CAGGTGTCAGTGCGTGTAGTAC
Ckb	Forward<br>Reverse	GCTCATTGACGACCACTTCCTC<br>CCTCCTCGTTAATCCACACCAG
Calcr	Forward<br>Reverse	AAGATGGACCCTCATGCCAGTG<br>CTCGTCGGTAAACACAGCCATG
Atp6v0d2	Forward<br>Reverse	ACGGTGATGTCACAGCAGACGT<br>CTCTGGATAGAGCCTGCCGCA
Mmp9	Forward<br>Reverse	GCTGACTACGATAAGGACGGCA<br>TAGTGGTGCAGGCAGAGTAGGA

Dc-stamp	Forward<br>Reverse	TTTGCCGCTGTGGACTATCTGC<br>GCAGAATCATGGACGACTCCTTG
Dc-stamp	Forward<br>Reverse	TTTGCCGCTGTGGACTATCTGC<br>GCAGAATCATGGACGACTCCTTG
Nfact1	Forward<br>Reverse	CAACGCCCTGACCACCGATAG<br>GGCTGCCTTCCGTCTCATAGT
Oc-stamp	Forward<br>Reverse	GGCTCAGAAGTTACCCACTGTC<br>GGAGGTTGGTTGAGGACGAAGA
Srebf1	Forward<br>Reverse	CGACTACATCCGCTTCTTGCAG<br>CCTCCATAGACACATCTGTGCC
Lss	Forward<br>Reverse	GCACACCACAGACCTGAGTTTC<br>CAGTGTGCTGAAGGAGAAACCAC
Plin2	Forward<br>Reverse	GACAGGATGGAGGAAAGACTGC<br>GGTAGTCGTCACCACATCCTTC
Hmgcs1	Forward<br>Reverse	GGAAATGCCAGACCTACAGGTG<br>TACTCGGAGAGCATGTCAGGCT
Slc25a1	Forward<br>Reverse	GGAGAGGACTATTGTGCGGTCT<br>CCCGTGGAAAAATCCTCGGTAC
Fads2	Forward<br>Reverse	TTCCTGGAGAGCCACTGGTTTG<br>GAAGAAGGACTGCTCCACATTGC
Scd1	Forward<br>Reverse	GCAAGCTCTACACCTGCCTCTT<br>CGTGCCTTGTAAGTTCTGTGGC
Scd2	Forward<br>Reverse	GTCTGACCTGAAAGCCGAGAAG<br>GCAAGAAGGTGCTAACGCACAG
Gapdh	Forward<br>Reverse	CATCACTGCCACCCAGAAGACTG<br>ATGCCAGTGAGCTTCCCGTTCAG
